# Jasmonates and Histone deacetylase 6 activate Arabidopsis genome-wide histone acetylation and methylation during the early acute stress response

**DOI:** 10.1186/s12915-022-01273-8

**Published:** 2022-04-11

**Authors:** Stacey A. Vincent, Jong-Myong Kim, Imma Pérez-Salamó, Taiko Kim To, Chieko Torii, Junko Ishida, Maho Tanaka, Takaho A. Endo, Prajwal Bhat, Paul F. Devlin, Motoaki Seki, Alessandra Devoto

**Affiliations:** 1 Plant Molecular Science and Centre of Systems and Synthetic Biology, Department of Biological Sciences, Royal Holloway University of London, Egham, Surrey, TW20 0EX UK; 2 Center for Sustainable Resource Science, RIKEN, 1-7-22 Suehiro-cho, Tsurumi, Yokohama, Kanagawa 230-0045 Japan; 3 Cluster for Pioneering Research, RIKEN, 2-1 Hirosawa, Wako, Saitama, 351-0198 Japan; 4 Present address: Ac-Planta Inc., 2-16-9 Yushima, Bunkyo-ku, Tokyo, 113-0034 Japan; 5 Graduate School of Agricultural and Life Sciences, The University of Tokyo, 1-1-1 Yayoi, Bunkyo-ku, Tokyo, 113-8657 Japan; 6 Present address: Department of Biological Sciences, The University of Tokyo, Tokyo, 113-0033 Japan; 7 Bioinformatics and Systems Engineering Division, RIKEN, 1-7-22 Suehiro-cho, Tsurumi, Yokohama, Kanagawa 230-0045 Japan; 8 Present address: Center for Integrative Medical Sciences, RIKEN, 1-7-22 Suehiro-cho, Tsurumi, Yokohama, Kanagawa 230-0045 Japan

**Keywords:** Arabidopsis, Abiotic, Biotic, Chromatin remodelling, Histone deacetylase, Histone modification, Jasmonate, Regulatory network, Specialized metabolism, Stress response

## Abstract

**Background:**

Jasmonates (JAs) mediate trade-off between responses to both biotic and abiotic stress and growth in plants. The *Arabidopsis thaliana* HISTONE DEACETYLASE 6 is part of the CORONATINE INSENSITIVE1 receptor complex, co-repressing the HDA6/COI1-dependent acetic acid-JA pathway that confers plant drought tolerance. The decrease in HDA6 binding to target DNA mirrors histone H4 acetylation (H4Ac) changes during JA-mediated drought response, and mutations in HDA6 also cause depletion in the constitutive repressive marker H3 lysine 27 trimethylation (H3K27me3). However, the genome-wide effect of HDA6 on H4Ac and much of the impact of JAs on histone modifications and chromatin remodelling remain elusive.

**Results:**

We performed high-throughput ChIP-Seq on the HDA6 mutant, *axe1-*5, and wild-type plants with or without methyl jasmonate (MeJA) treatment to assess changes in active H4ac and repressive H3K27me3 histone markers. Transcriptional regulation was investigated in parallel by microarray analysis in the same conditions. MeJA- and HDA6-dependent histone modifications on genes for specialized metabolism; linolenic acid and phenylpropanoid pathways; and abiotic and biotic stress responses were identified. H4ac and H3K27me3 enrichment also differentially affects JAs and HDA6-mediated genome integrity and gene regulatory networks, substantiating the role of HDA6 interacting with specific families of transposable elements in planta and highlighting further specificity of action as well as novel targets of HDA6 in the context of JA signalling for abiotic and biotic stress responses.

**Conclusions:**

The findings demonstrate functional overlap for MeJA and HDA6 in tuning plant developmental plasticity and response to stress at the histone modification level. MeJA and HDA6, nonetheless, maintain distinct activities on histone modifications to modulate genetic variability and to allow adaptation to environmental challenges.

**Supplementary Information:**

The online version contains supplementary material available at 10.1186/s12915-022-01273-8.

## Background

Transcriptional reprogramming, chromatin remodelling and crosstalk between phytohormone signalling pathways modulate plant adaptation to the environment [1–3]. Jasmonates (JAs) are oxylipins (oxygenated fatty acids) originating from linolenic acid [4–6], essential in mediating responses to pathogens and wounding [4, 5, 7, 8]. JAs regulate fertility, seed germination, reproduction and defence responses [9–14]. Drastic phenotypic alterations have been observed in planta linking JA to cell cycle and developmental processes [11, 12, 15].

JAZs (JASMONATE ZIM-DOMAIN proteins) that repress the JA response are targets for SCF^COI1^-mediated 26S proteasome degradation. The biologically active compound, (+)-7-iso-jasmonoyl-L-isoleucine (JA-Ile), facilitates the interaction between the SCF^COI1^ ubiquitin E3 ligase complex and JAZs via the F-box protein, CORONATINE INSENSITIVE1 (COI1) [16–19]. JAZs inhibit the activity of TFs like the basic helix-loop-helix (bHLH), MYC2, and its closest homologues, MYC3, MYC4 and MYC5 [16, 20–22], inducing metabolic changes [23]. The co- repressor, TOPLESS (TPL), is recruited by the JAZ ZIM domain (except in JAZ8) through the adaptor, Novel Interactor of JAZ (NINJA), also repressing JA responses [24–27].

The *Arabidopsis* histone deacetylase 6, HDA6/RPD3b (Reduced Potassium Dependency 3-like; namely HDA6), was originally identified as a repressor of transgene expression [28–30] and regulates gene activity and genome maintenance [3, 31]. HDA6 also silences some retrotransposon targets [19, 32]. HDA6 controls developmental processes [19, 31, 33–36] and stress responses in multiple plant models, being essential for cold, drought and salt stress tolerance [37–40]. HDA6 is also required for ABA-mediated responses to drought or salt [37, 41, 42]. JA signalling has long been associated with HDA6 function [39, 43, 44]. HDA6 is part of the COI1 receptor complex [43, 45] and associates with the JA response repressors JAZ3 and JAZ9 [46] and TPL [46, 47]. JAZ1 participates in this interaction which inhibits responses dependent on the ethylene- and JAs-responsive ETHYLENE-INSENSITIVE3 (EIN3) and ETHYLENE-INSENSITIVE3-like 1 (EIL1) transcription factors (TFs) [6, 33, 39, 46]. JAZ1 also interacts with MYC2 and MED25 (a subunit of the Mediator Complex, bridging transcription factors and RNA Pol lI bound to DNA) in the absence of JAs, and competitive binding of MED25 and JAZ9 for MYC3 follows JA-Ile elicitation [48, 49]. Kim et al. [39] demonstrated that the HDA6/COI1-dependent acetic acid-JA pathway confers plant drought tolerance and that ^14^C-labelled acetic acid, mediating drought response via the JA pathway, was incorporated into proteins with a molecular size corresponding to histone H4.

Chromatin remodelling regulates phytohormone-mediated plant stress responses including recent, albeit sparse, evidence on the JA response [3, 50–53]. Chromatin landscape modifications are governed antagonistically by histone deacetylases (HDACs) such as HDA6, and histone acetyltransferases (HATs), transferring acetyl groups from acetyl-CoA to histone Ks. Histone deacetylation represses genes and correlates with repressive chromatin remodelling markers of histone methylation such as histone H3 lysine 9 dimethylation (H3K9me2), histone H3 lysine 27 trimethylation (H3K27me3), and DNA methylation [34, 54–56]. HDACs also target transcription factors [57]. Chromatin methylation is mediated by the antagonistic methyltransferases and histone demethylases (HDMs) causing in mono-, di-, or trimethylated states of lysines [3, 58, 59]. In comparison to other histone markers, trimethylation of histone H3 Lys27 (H3K27me3) associates with transcriptionally silent states of the largest number of genes in *A. thaliana* [60].

HDA6 regulates modifications to histones 3 and 4 (H3; H4) [61]. HDA6-mediated regulation of H3 can induce specific transcriptional changes in long non-coding RNAs and regulate polyadenylation [62, 63]. The decrease in HDA6 binding can also mirror H4 acetylation (H4Ac) changes during JA-mediated drought response [39]. However, the genome-wide effect of HDA6 on H4Ac remains elusive. HDA6 deacetylates H4 at K5, K8 and K12, [31, 61]. Mutations in HDA6 alleles (*axe1-*5) also cause depletion in the constitutive repressive marker, H3K27me3 [31]. The role of chromatin remodelling in the JA response is just emerging [52, 53]. Moreover, HDA6-dependent JA regulation has yet to be uncoupled from the wider JA regulatory network and downstream targets of HDA6 on a genome-wide level are yet undescribed [6, 19]. The emergence of chromatin immunoprecipitation (ChIP-Seq) technology has allowed for the examination of chromatin remodelling on a genome-wide level [64, 65].

The early response to JAs associates with vast transcriptional reprograming in planta [15, 66–69]. In this study, specific genes undergoing histone modification and transcriptional induction in a MeJA and HDA6-dependent and independent manner have been discovered and linked to α-linolenic acid metabolism and phenylpropanoid biosynthesis through careful mining of public databases and literature. Functionally related clusters of MeJA- and HDA6-regulated genes have been identified and targeting of specific transcription factor families and transposable element superfamilies observed. Collectively, these discoveries show that MeJA-induced histone modifications modulate loci with roles in transcriptional regulation and specialized metabolism dependently or independently of HDA6, having therefore a potentially wider effect on chromatin remodelling. The comparison of our results with published studies [35, 62] also demonstrated that the role of HDA6 extends beyond its directly bound targets at the genome-wide level. The work provides novel targets and insight into both the role of JAs and the function of HDA6 that is part of the COI1 receptor complex and has important implications for the current understanding of JA-mediated plant development and stress responses.

## Results

### Genome-wide effects of methyl jasmonate and histone deacetylase 6 on specific histone markers and relationship with transcriptional regulation

To understand whether the chromatin landscape is modified by MeJA treatment and/or the histone deacetylase HDA6, genome-wide H4-tetra-acetylation (H4ac) and trimethylation at the 27th lysine residue of the histone H3 protein (H3K27me3) were analysed in whole *Arabidopsis* seedlings 12 days after sowing. Two key precedents have determined the focus of this work: the specific effect of the *axe1*-5 mutation on H4Ac during the JA- mediated drought response [39] and the effect of the *axe1*-5 mutation on the depletion in the constitutive repressive marker, H3K27me3 [31]. The *hda6*/*rpd3b* mutant, *axe1-*5, and its corresponding wild type, DR5, in the Col-0 background (namely wild type, WT) [29, 31, 39] were treated with methyl jasmonate (MeJA) or mock treated (-MeJA) and subjected to ChIP-Seq [70].

Anti-H4 tetra-acetylation antibodies, detecting acetylated lysine on H4 N-terminal tails (H4K5ac, H4K8ac, H4K12ac and H4K16ac), and anti-H3K27me3 antibodies, specific for histone H3 trimethylated at K27, were used [31]. These markers (namely H4ac and H3K27me3) were specifically chosen for their association with upregulation of stress-inducible genes in response to biotic and abiotic stresses [71] and with inactive genes, respectively, in both plants and animals [72]. Importantly, MeJA-inducible phenotypic plasticity has been previously shown in seedlings at this developmental stage [15].

**Fig. 1:**
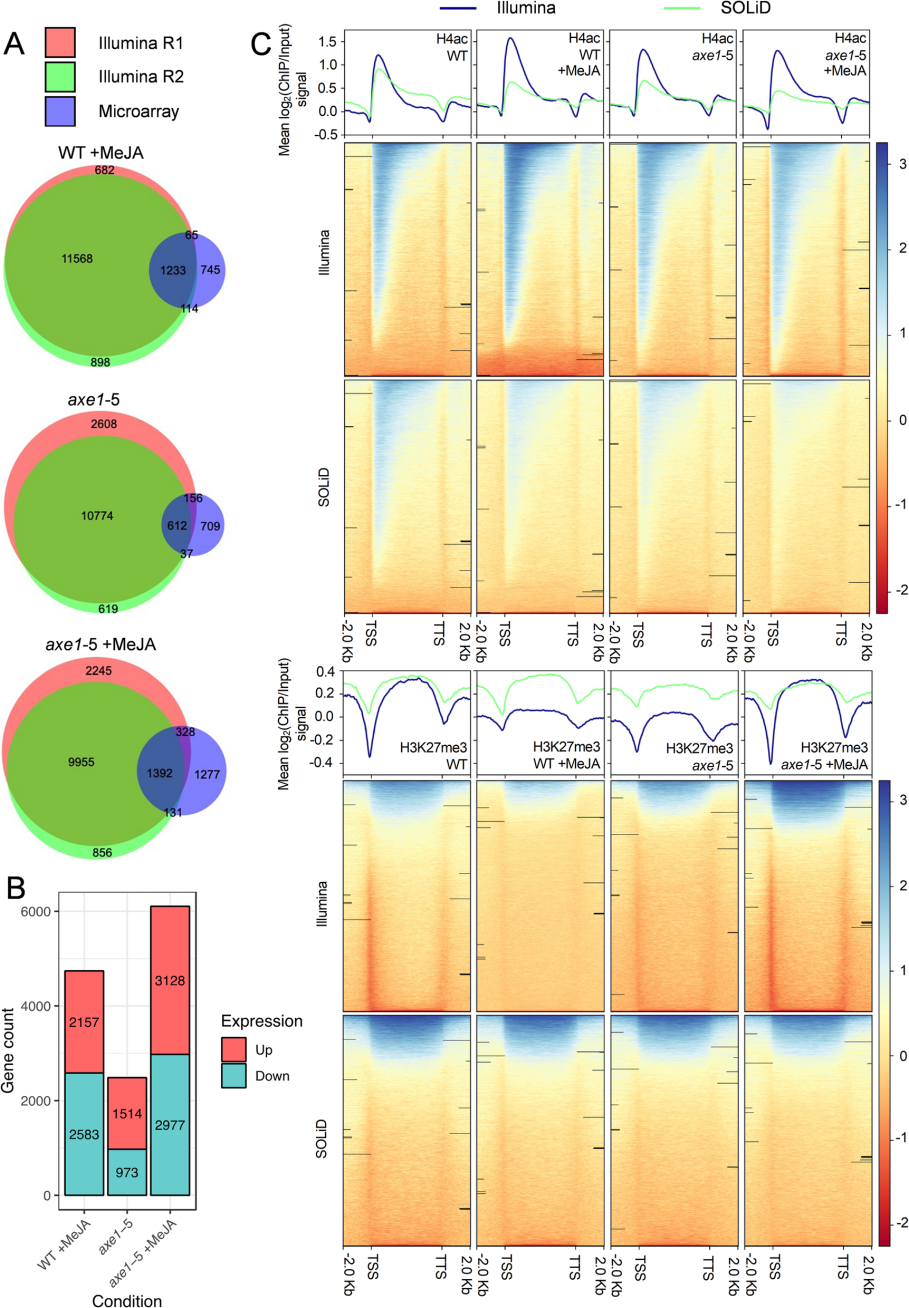
Histone markers enrichment across different platforms and loci. **A** Venn diagrams showing comparison between Illumina and microarray data for H4ac-associated genes. Comparison of H4ac-associated loci identified in three conditions of interest on different platforms: Illumina HiSeq replicates of ChIP-Seq data, and comparison of this data on the transcriptome level with microarray analysis. For Illumina, genes were considered to be significant in that condition if the peak summit generated by MACS was situated within identified loci. In the microarray data, genes with at least a 2-fold difference in their expression levels were evaluated with Student’s *t* test, and genes with *p*-values < 0.05 were counted as differentially expressed in the condition of interest. Diagrams created in BioVenn [73]. **B** Up- and downregulated genes identified by microarray analysis in three conditions of interest compared to the untreated WT control. **C** Histone marker enrichment distribution 2.0 kb upstream of transcription start sites (TSSs) and downstream of transcription termination sites (TTSs). The log2 ratio of ChIP signal to input on aligned files was calculated for both H4ac (upper panel) and H3K27me3 (lower panel) histone modifications using bamCompare from the deepTools2 package (Galaxy version 3.0.2.0) [74, 75] on WT and *axe1-*5-untreated and MeJA-treated samples. One representative replicate is shown

Sequencing reads across two biological replicates which were generated on the Illumina HiSeq platform were subjected to quality control and aligned to the TAIR10 reference genome to identify H4ac- and H3K27me3-enriched regions (Additional file 1: Figures S1-4). Three conditions of interest were defined: WT +MeJA (A), *axe1-5* (B) and *axe1-*5 +MeJA (C) to distinguish the effects of MeJA or HDA6. These conditions of interest were compared to the untreated WT to identify differentially enriched targets. For H4ac, 12801 (A), 11386 (B) and 11347 (C) gene-associated peaks, enriched in ChIP samples against the iVenn diagrams analysisnput, were reproducible in both biological replicates from the Illumina HiSeq (Fig. 1A) [70]. For H3K27me3, 1270, 2095 and 3444 gene-associated peaks were identified for WT +MeJA, *axe1-*5 and *axe1-*5 +MeJA, respectively, that were reproducible in both biological replicates from the Illumina HiSeq data (Additional file 1: Figure S5). An independent experiment with identical design was carried out on the SOLiD (Sequencing by Oligonucleotide Ligation and Detection) platform. Overall, the majority of the genes identified by SOLiD were also found with the Illumina analysis in the three conditions (Additional file 1: Figure S5).

The overlap between MeJA-induced transcripts and MeJA-induced histone acetylation was analysed. Transcription profiling, following the same experimental design as that used for ChIP-Seq, was performed on the same conditions of interest. Again, these were compared against the untreated WT control. Expression data on 28,897 genes within each sample was gained. Expression fold change was calculated between microarray datasets of WT +MeJA, *axe1-*5 untreated or *axe1-*5 +MeJA and WT untreated. Upregulated genes were compared to ChIP-Seq datasets for the H4ac [76] (Fig. 1A). For WT +MeJA, *axe1-*5 and *axe1-*5 +MeJA, 1233/2157 (57.2%), 612/1514 (40.4%), 1392/3128 (44.5%) common targets were identified, respectively, between upregulated transcripts and H4ac-enriched genes. Between downregulated transcripts and H3K27me3 enriched genes, 72/2583 (2.8%), 49/973 (5.0%) and 221/2977 (7.4%) common targets were identified for WT +MeJA, *axe1-*5 and *axe1-*5 +MeJA, respectively. A stronger relationship between gene-associated H4ac and MeJA transcriptional upregulation was, thus, observed. The MeJA-treated *axe1-*5 showed the highest number of up- and downregulated transcripts (Fig. 1B) whilst, conversely, the untreated *axe1-*5 had proportionally the fewest transcripts up- and downregulated.

Increased enrichment for the active histone marker H4Ac was observed at the transcription start site (TSS) and clear peaks for transcribed nucleosomes were observed directly downstream the TSS (Fig. 1C upper panel) with a signal drop at the nucleosome-free region upstream of the TSS. On the contrary, the signal for the repressive histone mark, H3K27me3, dropped sharply at the TSS and was enriched across the gene body (Fig. 1C, lower panel). The patterns were consistent between Illumina and SOLiD datasets. Genic regions were further broken down into 5′ UTR, exon, intron, 3′ UTR and intergenic, and signals corresponding to histone marker distribution were expressed as relative frequency with respect to TAIR 10 annotation for each condition (Additional file 1: Figure S6). Comparatively, H4ac and H3K27me3 histone modifications were more frequently distributed in the exons and introns, whereas marker distribution was reduced upstream of the TSSs, downstream of the TTSs and in the intergenic regions.

**Fig. 2:**
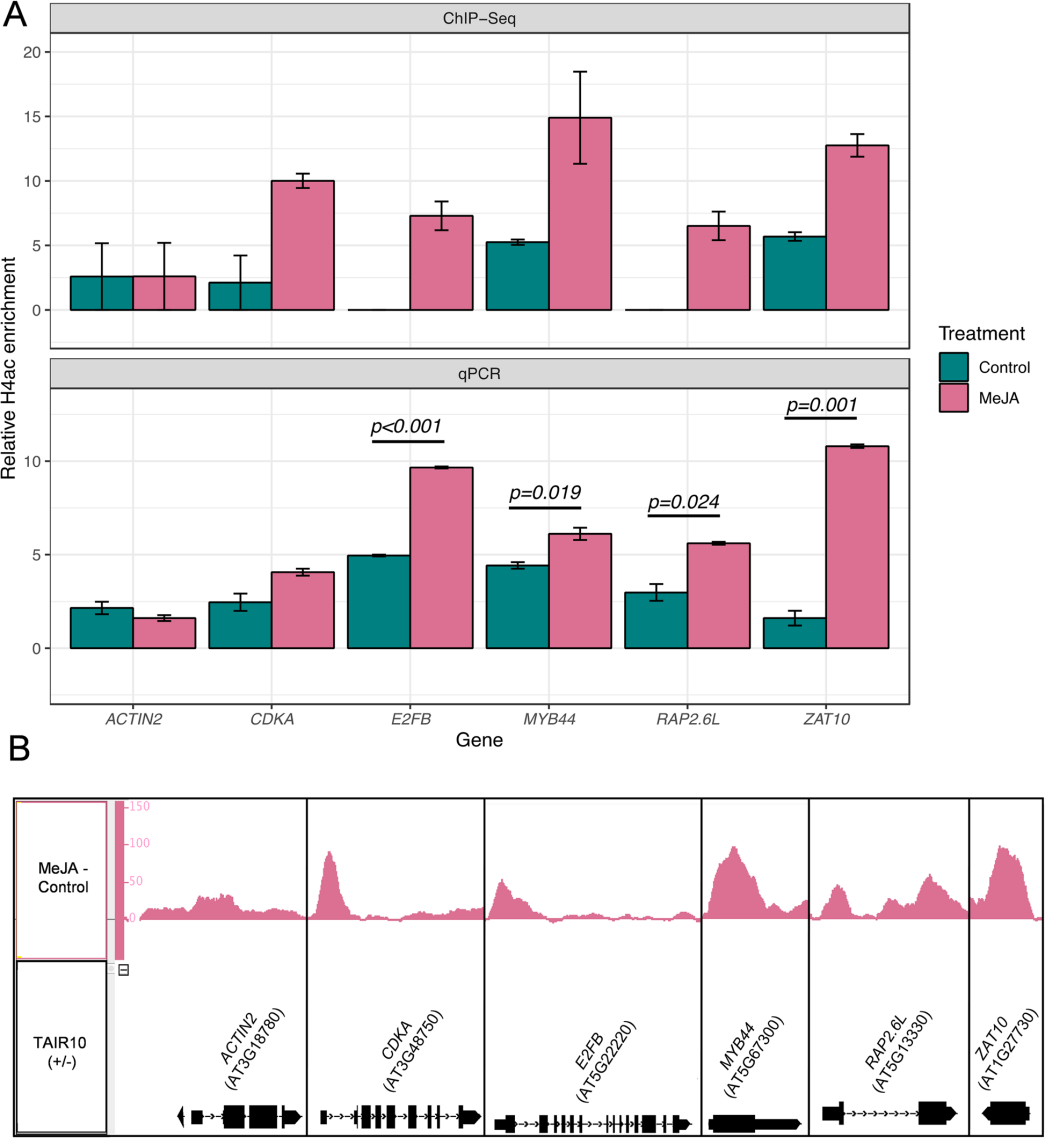
ChIP-quantitative polymerase chain reaction validation of ChIP-Seq targets enriched in H4ac. **A** ChIP-qPCR analysis for five genes identified with enriched H4ac following MeJA treatment plus one housekeeping gene (ACTIN2). Relative H4ac enrichment calculated as log2 fold change of H4ac as percentage of input against percentage input without H4ac antibody probing. ChIP-Seq enrichment against input for the same genes is also shown for comparison. Primers were designed in correspondence of the genomic regions most highly enriched in histone markers. Mean values and standard error of the mean (SEM) shown for three and two independent biological replicates for qPCR and ChIP-Seq analysis (Illumina), respectively. Statistical comparison of MeJA-treated and MeJA-untreated samples carried out on ChIP-qPCR data using Welch’s two-sample *t*-test. **B** H4ac-enriched ChIP-Seq peaks associated with genes of interest for one representative replicate. Visualized using Integrated Genome Browser [78]

Further independent experimental validation through ChIP-quantitative polymerase chain reaction (ChIP-qPCR) was carried out for selected genes on samples prepared in a separate H4ac ChIP experiment with the same experimental setup. MeJA signalling causes transcriptional reprogramming and cell cycle regulation [15, 67, 77]. Therefore, a subset of five known JA-responsive genes involved in the cell cycle, stress responses and defence responses were selected: *CYCLIN-DEPENDENT KINASE A* (*CDKA*; *AT3G48750*), *E2FB* (*AT5G22220*), *MYB DOMAIN PROTEIN 44* (*MYB44*; *AT5G67300*), *RELATED TO AP2 6L* (*RAP2.6L*; *AT5G13330*) and SALT TOLERANCE ZINC FINGER (STZ/*ZAT10*; *AT1G27730*). The findings from both ChIP-Seq and qPCR analysis were in agreement, displaying MeJA-induced H4ac enrichment consistently via both approaches (Fig. 2), with no distinct H4ac enrichment peak observed between MeJA-treated and untreated samples for the *ACTIN2* (*AT3G18780*) control gene.

**Fig. 3:**
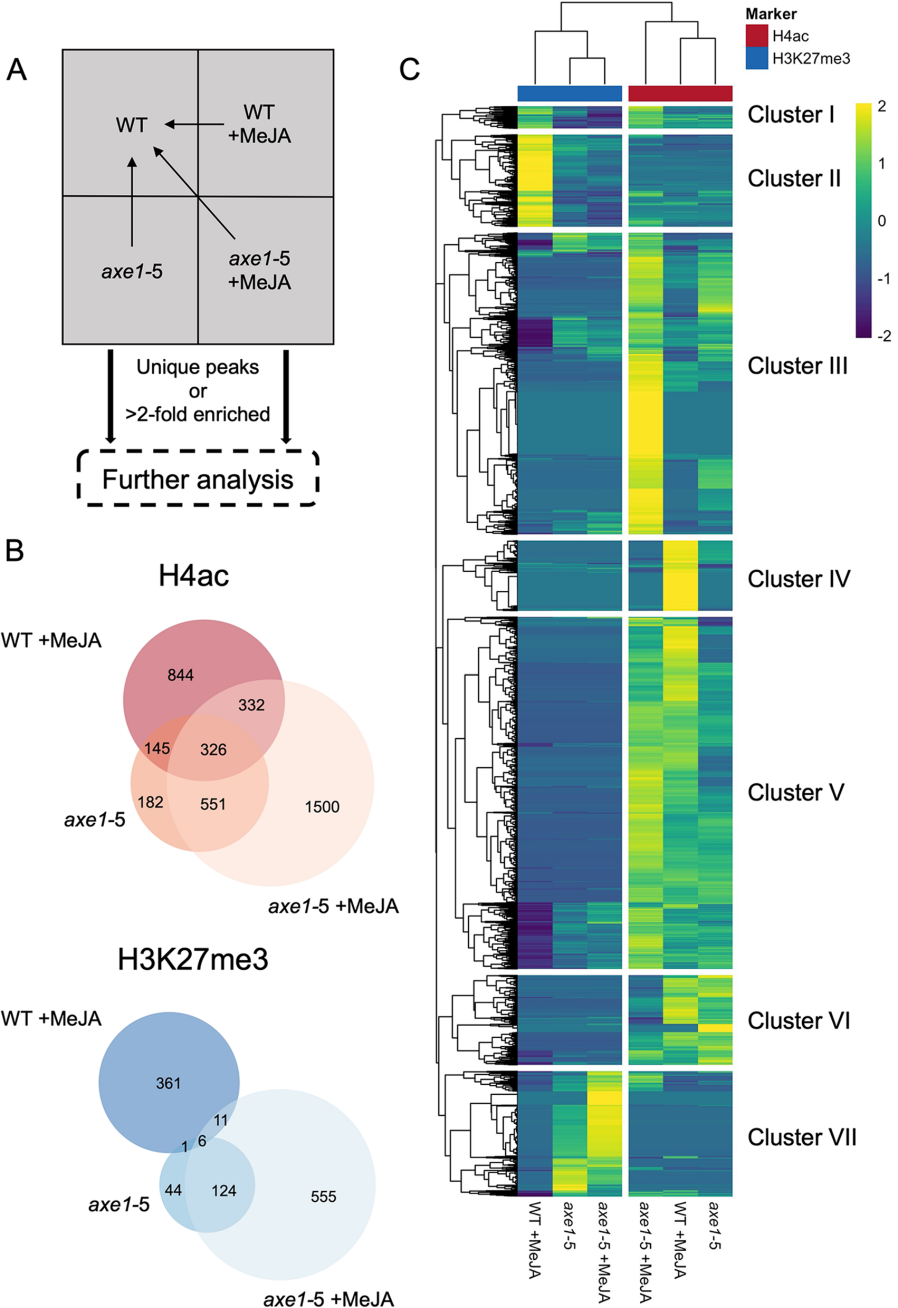
Gene-associated ChIP-Seq peaks in each condition of interest. **A** Workflow of peak enrichment from conditions of interest compared to untreated WT to identify unique or 2-fold enriched peaks before progressing with further analysis. **B** Venn diagrams showing number of genes differentially acetylated (H4ac) or methylated (H3K27me3) associated with conditions of interest (WT +MeJA, *axe1-*5 and *axe1-*5 +MeJA). Genes were determined to be differentially acetylated/methylated with respect to the untreated WT if the enrichment of the corresponding peak summit was at least ≥ 2 fold or where a peak associated with that gene was absent altogether in the untreated WT. *axe1-*5 +MeJA showed the greatest proportion of enriched genes: 69.82% (2709/3880) and 63.16% (696/1102) of the total for H4ac- and H3K27me3 respectively. The lowest proportion was associated with *axe1-*5: 31.03% (1204/3880) and 15.88% (175/1102) of the total for H4ac- and H3K27me3 respectively. Diagrams created in BioVenn [73]. **C** Heatmap and dendrogram showing hierarchical clustering of enrichment of genes associated with H4ac (red) and H3K27me3 (blue) in the three conditions. Clustering was performed using *k*-means clustering of Euclidean distances of differential enrichment values associated with each gene with respect to the untreated WT. Values were subsequently scaled by row to generate *Z*-scores of enrichment (see scale bar)

To distinguish the effect of MeJA treatment or loss of HDA6 function from ubiquitous histone modifications, signal-enriched genome regions were compared to the untreated WT and those either not present in the control or enriched more than twofold were selected for further analysis (Fig. 3A). The genes corresponding to the enriched signal were then considered to be regulated by MeJA or by the loss of HDA6 function. *axe1-*5 +MeJA showed widespread association of genes with both histone markers, whilst for untreated *axe1-*5 the association was limited. Overall, a higher proportion of genes was associated with H4ac modifications than H3K27me3, irrespective of the condition or genotype, showing that H4ac dynamics were more greatly affected by MeJA treatment and by HDA6 loss (Fig. 3B).

To analyse MeJA- or HDA6-dependent changes, the differences in enrichment values for H4ac or H3K27me3 for each gene, between the condition of interest and the untreated WT, were used to generate clusters based on k-means clustering of Euclidean distances using the complete linkage method (Fig. 3C). Broadly, both *axe1-*5-dependent conditions clustered most closely together with respect to H3K27me3 enrichment, whilst the untreated *axe1-*5 and WT +MeJA conditions showed more similarities in H4ac enrichment patterns. Seven main clusters were identified containing both common and specific targets of HDA6 and MeJA. Cluster I shows genes enriched in the repressive H3K27me3 marker in WT +MeJA, but also enriched in the activating H4ac marker in *axe1-*5 +MeJA. Cluster II showed similar H3K27me3 enrichment in WT +MeJA but limited H4ac enrichment in any condition of interest. Genes enriched in H4ac but not affected by any H3K27me3 enrichment in both *axe1-*5 conditions (Cluster III) and the WT +MeJA (Cluster IV) condition were also identified. Remarkably, genes in cluster V were enriched in H4ac in all three conditions, highlighting potential common targets of HDA6 and MeJA-mediated histone modifications. Common targets of H4ac enrichment in the untreated *axe1-*5 and WT +MeJA that were not enriched in *axe1-*5 +MeJA (Cluster VI) were also observed, as well as *axe1-*5-dependent H3K27me3 enrichment (Cluster VII).

We compared our dataset to published studies [35, 62] to identify the overlap of Arabidopsis genes enriched in H4ac and H3K27me3 in *axe1*-5 in our study with those enriched in H3 acetylation (H3ac) or H3 lysine 4 dimethylation (H3K4me2) in WT versus an *hda6* mutant based on the ChIP-Seq by Hung et al. [35, 62, 79, 80]. The analysis showed that the highest overlap occurred, unsurprisingly, between the active markers H4Ac (our dataset) and H3Ac and H3K4me2 [62, 80] with the average overlap between conditions being 12.36% and 5.07%. Furthermore, the overlap between the HDA6 binding [35, 79] and the H4Ac and H3K27me3 markers used in our study was only 0.99% and 1.01%. Our analysis of the overlap between the HDA6 binding and the H3ac and H3K4me2 markers from Hung et al [35, 62, 79, 80], showed a similar overlap of 1.56% and 1.21% respectively, strengthening the validity of our findings (Additional file 2: Table S3).

Altogether, the comparisons between conditions through clustering and Venn diagrams analysis demonstrated that the *axe1-*5 +MeJA condition has more unique targets enriched in H4Ac than either the WT +MeJA or *axe1-*5 conditions. Conversely, for H3K27me3, the fact that MeJA-treated and MeJA-untreated *axe1-*5 are clustering more closely with each other than with WT +MeJA, shows the important effect of HDA6 on the enrichment for this marker.

### Methyljasmonate- and histone deacetylase 6-mediated H4ac and H3K27me3-enrichment associate with distinct pathways

**Fig. 4:**
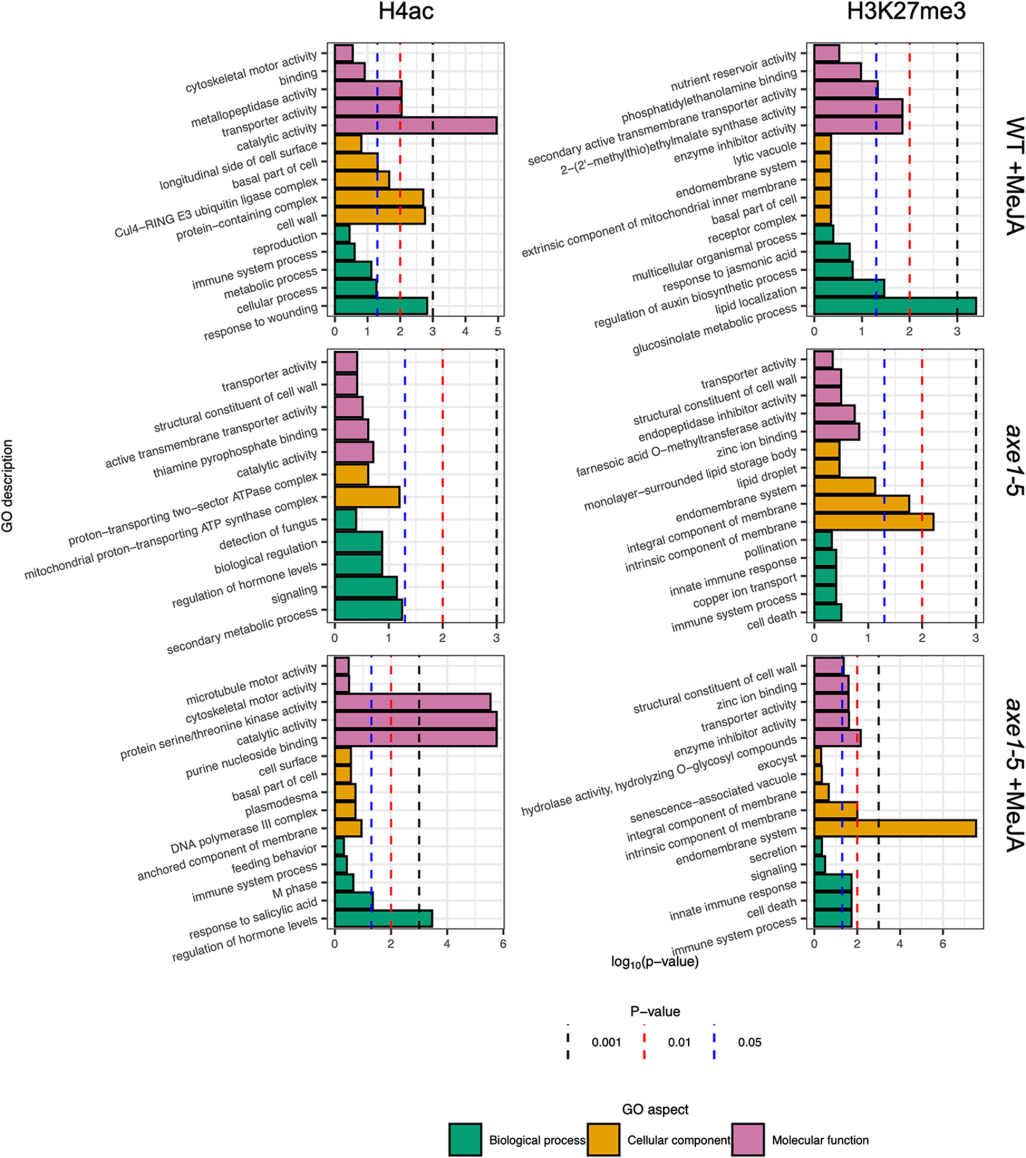
Gene Ontology (GO) terms associated with genes enriched in H4ac and H3K27me3. **A** Statistically overrepresented GO terms for the five least dispensable terms for Biological Process, Cellular Component and Molecular Function. −log10 (*p*-value) for the conditions of interest (WT +MeJA, *axe1-*5 and *axe1-*5 +MeJA). Statistical analysis was carried out in the Cystoscape v3.6.1 BiNGO plugin using a hypergeometric test corrected for Benjamini and Hochberg False Discovery Rate (FDR) [81, 82]. Redundant GO terms were consolidated and dispensability for each term was calculated using REVIGO [83]. Dashed lines indicate statistical significance of the *p*-value

Our observations above were also corroborated by pathway analysis using GO and KEGG databases. To gain initial functional insights on the possible role of H4ac or H3K27me3 enrichment, gene ontology (GO) analysis was performed using Cytoscape (v3.6.1) and the BiNGO plugin [81, 82]. Overrepresented ontology terms were grouped according to Biological Process (BP), Molecular Function (MF) and Cellular Component (CC). Redundant GO terms were consolidated and dispensability for each term was calculated using REVIGO [83] (Fig. 4; Table 1). Other highly overrepresented BP GO categories for H4ac were indole glucosinolate biosynthetic process, allene oxide cyclase activity and DNA polymerase III complex for WT +MeJA, *axe1-5* and *axe1-*5 +MeJA respectively (Additional file 2: Table S4). Associated with H3K27me3 were stamen filament development for WT +MeJA and sucrose transport for *axe1-5* and *axe1-*5 +MeJA, indicating that the two histone modifications affect distinct targets, consistent with their opposing functional roles. For H3K27me3, the overrepresented categories ‘cell death’, ‘immune system process’ and ‘innate immune response’ were common between *axe1-*5 +MeJA and *axe1-*5, albeit not significant in the latter (Fig. 4, Additional file 1: Figure S4). Within these categories, ~92% of the total number of genes enriched for H3K27me3 show enrichment dependent on HDA6 whilst for ~71%, the enrichment in the *axe1-*5 background is MeJA dependent. Overall, the *axe1-*5 +MeJA and *axe1-*5 conditions show the largest overlap in terms of genes targeted by the two histone modifications, strengthening the relatedness observed through clustering by enrichment (Fig. 3B). Notably ~45% of the genes targeted by H3K27me3 in the overrepresented categories ‘cell death’, ‘immune system process’ and ‘immune response’, are also enriched for H4ac, suggesting preferential histone modification at specific genes for disease resistance proteins of the TIR-NBS-LRR class [84]. Previous studies have shown that HDA6 (*shi5* allele) represses the expression and H3Ac enrichment of several pathogen defence-responsive genes [85]. A direct comparison of the genes in the KEGG pathways category ‘Plant-pathogen interaction’, enriched in H4ac in the *axe1-*5 +MeJA background, with the genes identified by Wang et al. [85], did not show any extended overlap (d.n.s.), highlighting, therefore, histone marker- and allele-specific effects on genes involved either in generic defence responses or molecular plant-pathogen interaction.

**Table 1:** KEGG pathways overrepresented by H4ac- and H3K27me3-associated AGIs. Statistically significant pathways shown

Histone modification	KEGG ID	Pathway description	Condition								
			WT +MeJA			*axe1-*5			*axe1-*5 +MeJA		
			No. AGIs in condition	Percentage representation (%)	*p*-value (Fisher’s exact)	No. AGIs in condition	Percentage representation (%)	*p*-value (Fisher’s exact)	No. AGIs in condition	Percentage representation (%)	*p*-value (Fisher’s exact)
H4ac	KO02010	ABC transporters	–	–	–	–	–	–	8	0.3	2.000E−04
	KO00250	Alanine, aspartate and glutamate metabolism	8	0.5	1.700E−02	–	–	–	–	–	–
	KO00592	alpha-Linolenic acid metabolism	8	0.5	2.900E−03	–	–	–	7	0.3	1.400E−02
	KO00330	Arginine and proline metabolism	–	–	–	–	–	–	8	0.3	3.700E−02
	KO00410	beta-Alanine metabolism	–	–	–	–	–	–	7	0.3	2.400E−02
	KO01110	Biosynthesis of secondary metabolites	87	5.3	2.100E−02	–	–	–	115	4.3	1.100E−07
	KO00710	Carbon fixation in photosynthetic organisms	10	0.6	2.100E−02	–	–	–	–	–	–
	KO00904	Diterpenoid biosynthesis	–	–	–	4	0.3	7.600E−03	6	0.2	4.000E−03
	KO00062	Fatty acid elongation	–	–	–	–	–	–	8	0.3	2.500E−03
	KO00941	Flavonoid biosynthesis	5	0.3	1.300E−02	–	–	–	6	0.2	3.100E−03
	KO00950	Isoquinoline alkaloid biosynthesis	5	0.3	1.900E−02	–	–	–	7	0.3	9.300E−04
	KO01100	Metabolic pathways	–	–	–	–	–	–	159	5.9	2.800E−03
	KO00040	Pentose and glucuronate interconversions	–	–	–	7	0.6	2.000E−02	13	0.5	3.100E−03
	KO00360	Phenylalanine metabolism	–	–	–	–	–	–	11	0.4	1.500E−04
	KO00940	Phenylpropanoid biosynthesis	–	–	–	–	–	–	23	0.9	9.300E−04
	KO04075	Plant hormone signal transduction	–	–	–	18	1.5	7.600E−03	27	1.0	4.700E−02
	KO04626	Plant-pathogen interaction	–	–	–	–	–	–	19	0.7	2.200E−02
	KO00240	Pyrimidine metabolism	13	0.8	4.900E−02	–	–	–	–	–	–
	KO03008	Ribosome biogenesis in eukaryotes	13	0.8	2.300E−02	–	–	–	–	–	–
	KO00430	Taurine and hypotaurine metabolism	–	–	–	–	–	–	4	0.1	1.600E−02
	KO00960	Tropane, piperidine and pyridine alkaloid biosynthesis	–	–	–	–	–	–	7	0.3	1.400E−02
	KO00380	Tryptophan metabolism	–	–	–	–	–	–	10	0.4	1.500E−03
	KO00350	Tyrosine metabolism	7	0.4	2.000E−02	–	–	–	10	0.4	4.500E−04
	KO00130	Ubiquinone and other terpenoid-quinone biosynthesis	6	0.4	3.300E−02	–	–	–	7	0.3	1.200E−02
	KO00908	Zeatin biosynthesis	5	0.3	1.100E−02	–	–	–	7	0.3	3.600E−04
H3K27me3	KO02010	ABC transporters	–	–	–	–	–	–	3	0.4	4.600E−03
	KO00460	Cyanoamino acid metabolism	3	0.8	1.700E−02	–	–	–	5	0.7	1.600E−03
	KO00040	Pentose and glucuronate interconversions	4	1.1	4.700E−03	–	–	–	–	–	–
	KO00940	Phenylpropanoid biosynthesis	–	–	–	–	–	–	7	1.0	6.900E−03
	KO04075	Plant hormone signal transduction	–	–	–	5	2.9	3.200E−03	10	1.4	4.200E−03
	KO00909	Sesquiterpenoid and triterpenoid biosynthesis	3	0.8	1.100E−03	–	–	–	–	–	–
	KO00500	Starch and sucrose metabolism	–	–	–	–	–	–	7	1.0	1.900E−03
	KO00380	Tryptophan metabolism	3	0.8	8.100E−03	–	–	–	–	–	–

**Fig. 5:**
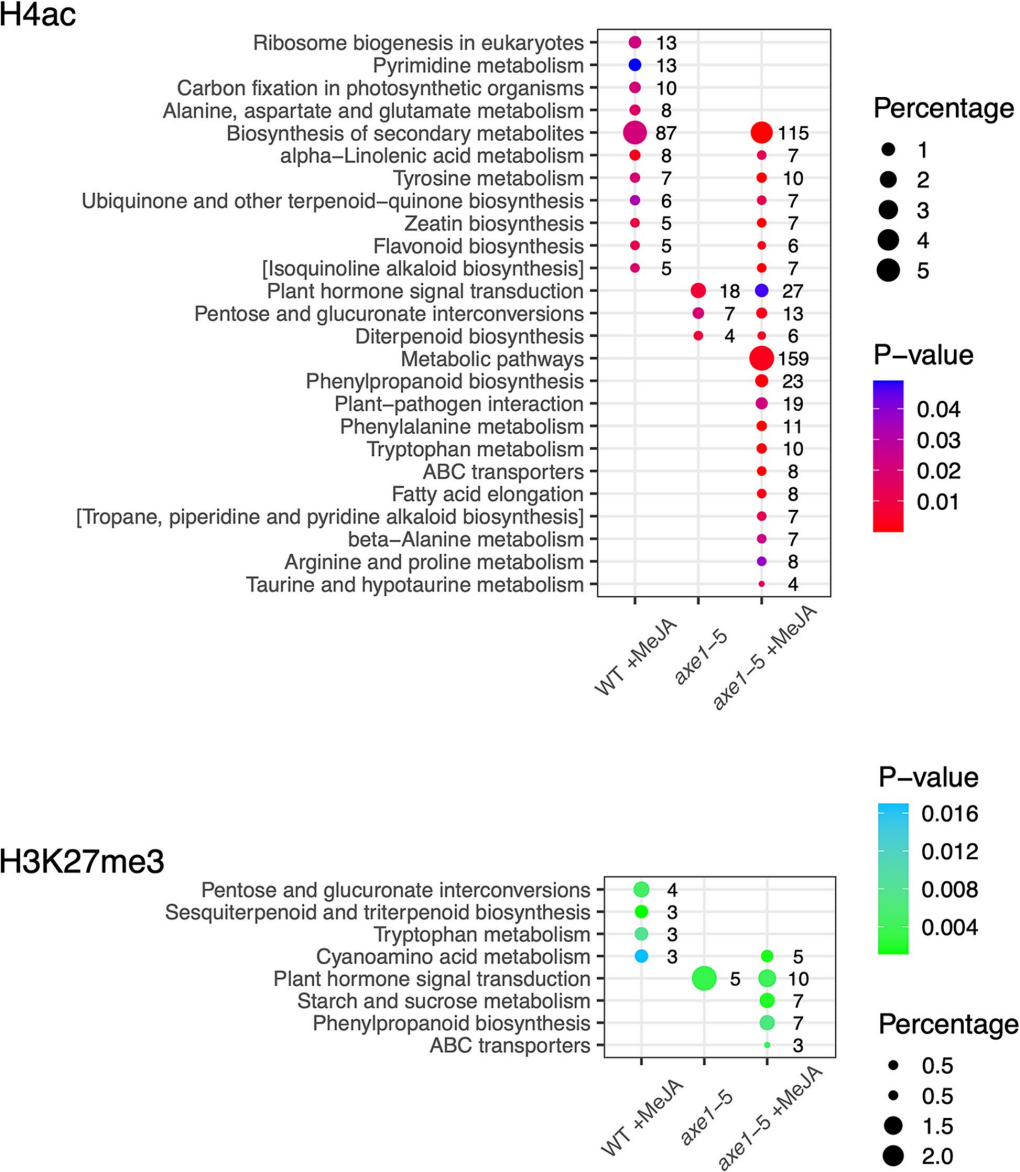
Overrepresentation of KEGG pathways represented by genes enriched in H4ac and H3K27me3. Overrepresented KEGG pathways and their statistical significance (*p*-value), % coverage with respect to total number of genes in a pathway and raw gene count for H4ac- (**A**) and H3K27me3-associated genes (**B**) for the conditions of interest (WT +MeJA, *axe1-*5 and *axe1-*5 +MeJA). The statistical analysis for pathway overrepresentation was carried out in DAVID using Fisher’s exact test [86, 87]

Complementary GO analysis using DAVID [86, 87] identified overrepresented biochemical pathways associated with H4ac and H3K27me3, in the three conditions. The highest number of overrepresented KEGG pathways was associated with *axe1-*5 +MeJA. The following analysis has been complemented by careful examination of literature to avoid drawing assumptions based on general hits, derived from database automation, on categories not defined in Arabidopsis. The categories overrepresented in *axe1-5* were all shared with *axe1-*5 +MeJA for both histone markers. In *axe1-*5 +MeJA, the most significant KEGG pathway, with the highest percentage representation, was ‘Metabolic pathways’ for H4ac (Fig. 5; Table 1). ‘Biosynthesis of secondary metabolites’ was overrepresented in both WT +MeJA and *axe1-*5 +MeJA, also for this marker, indicating MeJA action independent of HDA6 regulating specialized metabolism, albeit specifically for this general group. Notably, α-Linolenic acid metabolism overrepresentation became significant after MeJA treatment, and ‘ribosome biogenesis’ ranked the highest among the categories unique to the WT +MeJA for H4ac.

**Fig. 6:**
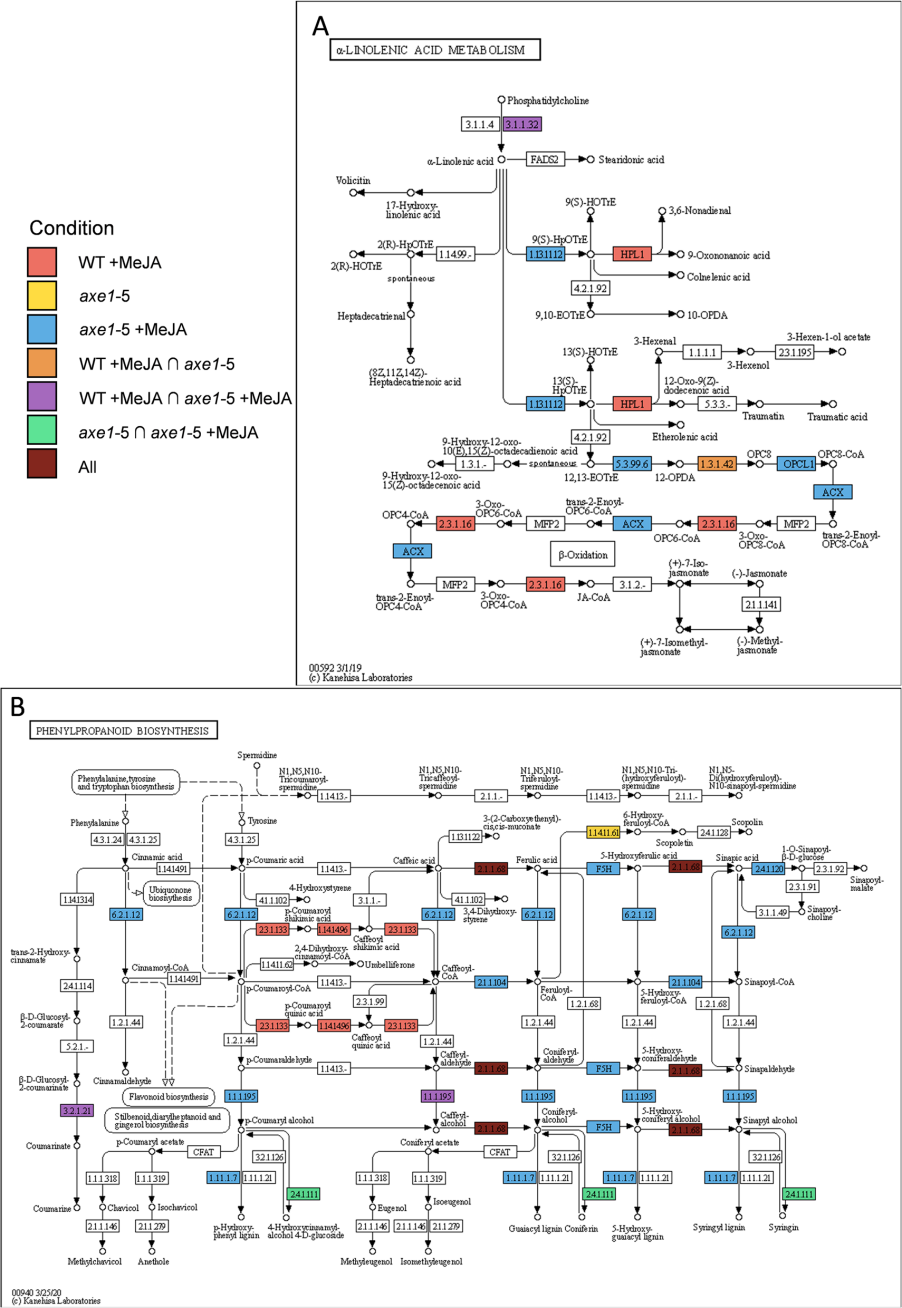
Mapping genes enriched in H4ac to secondary metabolism nodes. Mapping details for the **A** α -linolenic acid metabolism (α-LA) and **B** phenylpropanoid (PP) biosynthesis showing that MeJA caused the enrichment of the activating marker, H4ac, on specific genes for α-LA biosynthetic enzymes in a HDA6-dependent or HDA6-independent manner. In the first category were hydroperoxide lyase (HPL1; AT4G15440) and acetyl-CoA acyltransferase 1/peroxisomal 3-ketoacyl-CoA thiolase (PKT4; AT1G04710) (WT +MeJA). The H4ac enrichment of the phospholipase A1 (DAD1, AT2G44810); lipoxygenases LOX2 (AT3G45140) LOX3 (AT1G17420), LOX4 (AT1G72520), LOX6 (AT1G67560), allene oxide cyclases AOC1/2/3 (AT3G25760, AT3G25770, AT3G25780), and 12-oxophytodienoic acid reductase OPR2 (AT1G76690) was instead HDA6 independent. For PP, the activating marker was enriched by MeJA in WT, but not in an *axe1-*5 background, namely on hydroxycinnamoyl-CoA shikimate/quinate hydroxycinnamoyl transferase (HCT, AT5G48930) and CYP98A3 (AT2G40890). The H4ac enrichment of most enzymes in the pathway was instead *axe1-*5 independent. The following genes were specifically enriched: the ferulate-5-hydroxylase FAH1 (AT4G36220), the UDP-Glycosyltransferase UGT84A2 (AT3G21560); several 4-coumarate:CoA ligases (4CL1/2/3, AT1G51680, AT3G21240, AT1G65060); a member of the S-adenosyl-L-methionine-dependent methyltransferases superfamily (AT4G26220); FAD-binding Berberine family protein member, cinnamyl-alcohol dehydrogenases (AT1G30760) and ELI3-1 (cinnamyl-alcohol dehydrogenase 7 (AT4G37980). Several members of the peroxidase superfamily linked to phenylpropanoid/monolignols biosynthesis and stress responses [89, 90] were also enriched: PR9 (AT1G05240), PRX2 (AT1G05250), RCI3 (RARE COLD INDUCIBLE GENE 3; cationic peroxidase; AT1G05260) and several uncharacterized ones (AT2G18150, AT5G14130, AT5G17820, AT5G58390 and AT5G58400). Colours as described in the International Union of Biochemistry and Molecular Biology (IUBMB) enzyme nomenclature shown

iPath explorer [88] produced a high-level bird’s-eye overview of the Arabidopsis metabolism associated with changes in H4ac at specific genes for the three conditions (Additional file 1: Figure S7). As expected, *axe1-*5 +MeJA shows the highest enrichment of metabolism-associated genes (~35% of which are unique to this condition). WT +MeJA had the second highest number of metabolism-associated genes (~24% unique to this condition). MeJA treatment, therefore, has the predominant effect on regulating the abundance of the activating histone marker on metabolism-related genes and the effect of *axe1-*5 genotype alone does not mirror the effect of MeJA treatment. This is in line with the overrepresentation of the KEGG pathways (Fig. 5). For example, the genes corresponding to enzymes in the modules, ‘α-Linolenic acid metabolism’ and ‘Phenylpropanoid biosynthesis’, are differentially enriched for H4ac in each condition (Fig. 6A, B) but, remarkably, MeJA caused the enrichment of H4ac on specific genes for these biosynthetic enzymes in an HDA6-dependent and HDA6-independent manner respectively. The KEGG category ‘Phenylpropanoid biosynthesis’ was unique to *axe1-*5 +MeJA for both histone markers (Fig. 6B).

Among the KEGG categories associated with the *axe1-*5 genotype (+/-MeJA) was ‘Plant hormone signal transduction’ (Fig. 5). This was significantly overrepresented in association with H4ac in an *axe1-5* derepressed background in the presence or absence of MeJA (Fig. 5). Interestingly, many genes in this ‘Plant hormone signal transduction’ category that are enriched in H4ac and dependent either on HDA6 or MeJA action are associated with ‘auxin response’, such as GH3 family and SAUR-like proteins [91, 92]. Our study extends the crosstalk paradigm further by including the effect of HDA6 and providing novel targets for analysis with respect to what previously reported [93–95].

Also enriched in H4ac in the *axe1-*5 background were regulatory components of abscisic acid (ABA) receptor genes (PYL2) [96], the ABA-induced PP2C protein 3 (HAI3) [96], the ethylene responsive element binding factor 2 (ERF2), the GA-INSENSITIVE DWARF1a (GID1a) receptor component activator of the gibberellic acid (GA) response [97] and JAZ6 (as the only JAZ family member; Additional file 3: Supplementary Data Table). Looking at other JAZ MeJA signalling components, JAZ4 was enriched for H3K27me3 only in *axe1-*5 +MeJA. JAZ8 was the only JAZ family member enriched in H4ac following MeJA treatment in WT or in the *axe1-*5 mutant untreated, the latter condition being in common with the condition in which JAZ6 was enriched. Overall, these findings also demonstrate that MeJA has a more prominent role than HDA6 in regulating H4Ac modification on enzymes for specialized metabolism.

### Methyljasmonate and axe1-5 mediate changes of H4ac and H3K27me3 on mobile genetic elements

The activity of transposable elements (TEs) can lead to chromatin remodelling and affect transcriptional regulation in plants [98]. Environmental stress can reactivate TEs suppressed by plant genome-defence mechanisms [99]. Specific retrotransposons, like ATCOPIA, ATLINE1-4, ATLANTYS, ATGP1 and Sadhu, are suppressed by HDA6 [31, 100–103]. We investigated here, in high-throughput, the genome-wide effect of *axe1-*5 and MeJA on TEs. TE-associated loci were deemed to be differentially enriched in H4ac or H3K27me3 if ChIP-Seq peaks were only present in WT +MeJA, *axe1-*5 and *axe1-*5 +MeJA, or twofold enriched in WT +MeJA, *axe1-*5 and *axe1-*5 +MeJA when compared to the untreated WT.

**Fig. 7:**
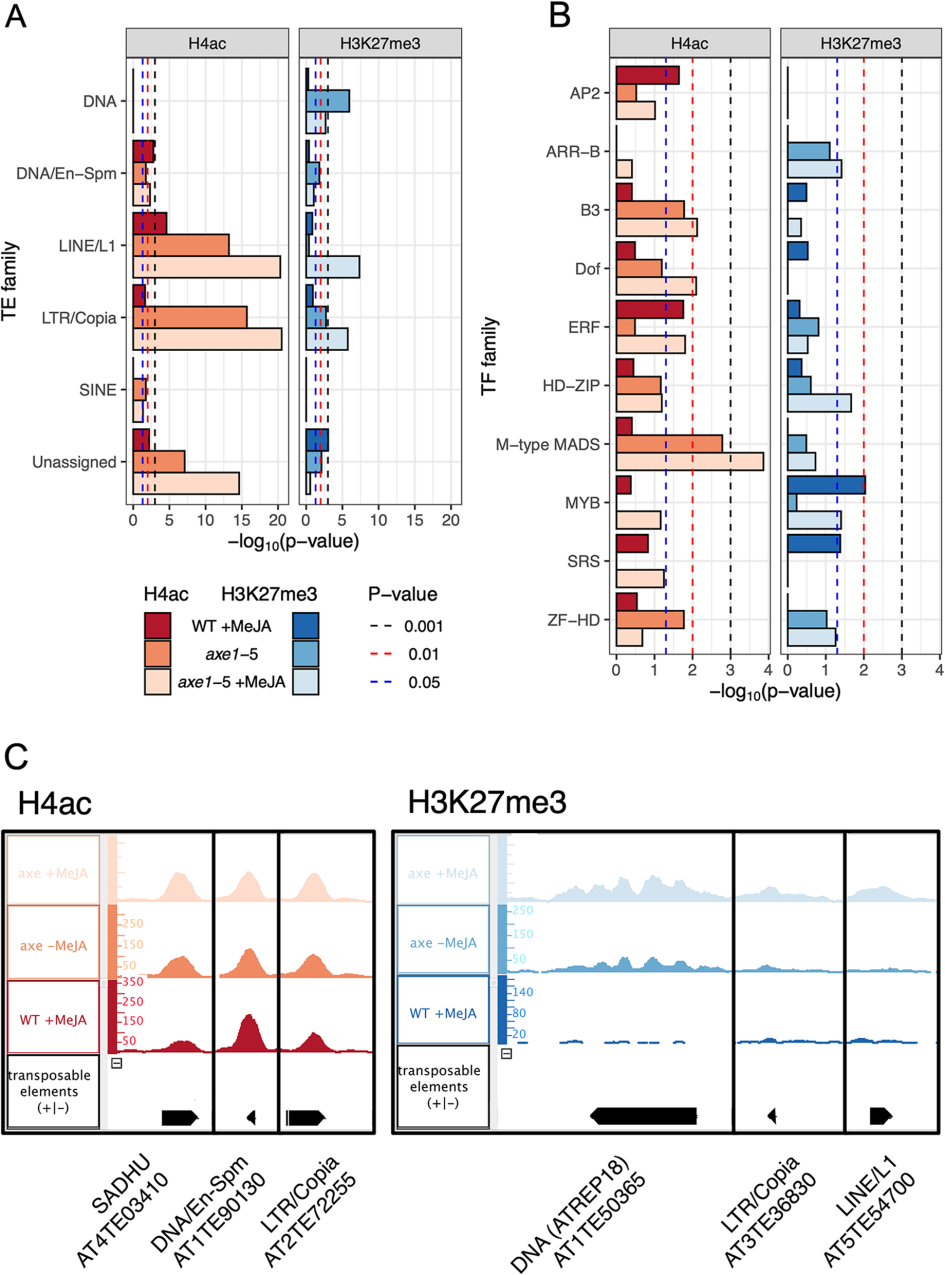
Overrepresentation of transcription factor or transposon families associated with H4ac or H3K27me3 for WT +MeJA, *axe1-*5 and *axe1-*5 +MeJA. **A** Overrepresented transposable element superfamilies extracted from TAIR10. **B** Overrepresented transcription factor families identified using PlantTFDB [104]. Families and superfamilies shown are significant in at least one condition. Statistical analysis was performed using a hypergeometric test. **C** H4ac- (left) and H3K27me3-enriched (right) ChIP-Seq peak examples associated with transposable elements for one representative replicate. Visualized using Integrated Genome Browser [78]

There are 31,189 annotated TEs in Arabidopsis, belonging to over 18 superfamilies of which five are overrepresented in at least one condition for either histone marker: DNA, DNA/En-Spm, LINE (long interspersed elements)/L1, LTR (long terminal repeat)/COPIA and SINE (short interspersed elements) (Fig. 7A). The DNA/En-Spm, LINE/L1 and LTR/COPIA superfamilies were significantly overrepresented in all three conditions for H4ac (Table 2). SINE was overrepresented in the *axe1-*5 and *axe1-*5 +MeJA conditions. None of these superfamilies was overrepresented in WT +MeJA in association with H3K27me3, though. DNA (mostly members of the ATREP18 family) and LTR/COPIA (including AT4TE06710 and AT3TE76225) were overrepresented in both *axe1-*5 and *axe1-*5 +MeJA in association with H3K27me3, whereas DNA/En-Spm and LINE were overrepresented in *axe1-*5 and *axe1-*5 +MeJA respectively. Of the TEs not assigned to any superfamily, five of the possible 16 TEs in the Sadhu family were enriched for H4ac in *axe1-*5 +MeJA (AT3TE88640, AT3TE60310, AT3TE63935, AT5TE36235 and AT4TE03410). The latter four were also enriched in *axe1-*5 indicating a specific role for the genotype for the regulation of the Sadhu TEs via H4ac. Overall, whilst the *axe1-*5 genotype has a greater effect on H4ac-mediated regulation of TEs, MeJA alone caused a specific enrichment for TEs within the DNA/En-Spm, LINE/L1 and LTR/COPIA families. The enrichment of H3K27me3 on LINE/L1 TEs was only overrepresented in *axe1-*5 +MeJA in contrast to H4ac-associated LINE/L1 TEs which were overrepresented in all three conditions. These findings demonstrate novel differential associations of both the active and repressive histone modifications, as well as of the roles of HDA6 and MeJA in TE regulation.

**Table 2:** Overrepresentation of transposable element superfamilies in H4ac- and H3K27me3-associated loci. Only statistically overrepresented families shown

Histone marker	TE Superfamily	No. TEs in *A. thaliana*	Condition								
			WT +MeJA			*axe1-*5			*axe1-*5 +MeJA		
			No. of TEs in condition	Percentage representation (%)	*p*-value (Hypergeometric test)	No. of TEs in condition	Percentage representation (%)	*p*-value (Hypergeometric test)	No. of TEs in condition	Percentage representation (%)	*p*-value (Hypergeometric test)
H4ac	DNA	1829	0	0.0	NSS	1	0.1	NSS	1	0.1	NSS
	DNA/En-Spm	941	5	0.5	1.61E−03	9	1.0	1.80E−02	13	1.4	4.82E−03
	LINE/L1	1366	8	0.6	2.50E−05	30	2.2	6.07E−14	45	3.3	4.94E−21
	LTR/Copia	1781	5	0.3	2.28E−02	37	2.1	2.03E−16	51	2.9	3.15E−21
	SINE	131	0	0.0	NSS	3	2.3	1.80E−02	3	2.3	4.51E−02
	Unassigned	129	2	1.6	6.41E−03	8	6.2	7.77E−08	15	11.6	2.35E−15
	Total	31,189	20			88			128		
H3K27me3	DNA	1829	3	0.2	NSS	11	0.6	1.12E−06	12	0.7	2.11E−03
	DNA/En-Spm	941	2	0.2	NSS	4	0.4	1.51E−02	5	0.5	NSS
	LINE/L1	1366	4	0.3	NSS	2	0.1	NSS	17	1.2	4.43E−08
	LTR/Copia	1781	5	0.3	NSS	7	0.4	1.86E−03	17	1.0	1.83E−06
	SINE	131	0	0.0	NSS	0	0.0	NSS	0	0.0	NSS
	Unassigned	129	3	2.3	9.21E−04	2	1.6	7.76E−03	1	0.8	NSS
	Total	31,189	17			26			52		

### Methyljasmonate- and histone deacetylase 6 cause differential enrichment of H4ac and H3K27me3 on transcription factors families

To investigate the effect of JA-mediated histone modifications on transcriptional reprogramming and identify hubs for gene regulatory networks (GRNs) and potential chromatin remodelling [105], the representation of transcription factor families was examined for H4ac and H3K27me3 enrichment. Available and relevant genome-wide expression studies [49, 53, 69] have been analysed in parallel to detect possible overlaps (d.n.s.). There are 1717 annotated transcription factors in Arabidopsis belonging to over 19 TF families [104]: 10 families were significantly overrepresented in at least one condition for either histone marker: APETALA2 (AP2), ARABIDOPSIS RESPONSE REGULATOR-B (ARR-B), B3 DOMAIN (B3), DNA-binding with one finger (Dof), ETHYLENE RESPONSE FACTOR (ERF), HOMEODOMAIN-LEUCINE ZIPPER (HD-ZIP), M-type (MCM1, AGAMOUS, DEFICIENS, and SRF, serum response factor) MADS-domain, MYB domain, SERYL-TRNA SYNTHETASE (SRS), ZINC FINGER HOMEODOMAIN (ZF-HD) (Fig. 7B; Table 3). The combination of MeJA and *axe1-*5 positively affects the H4Ac enrichment of TF families. The enrichment in H3K27me3 was, overall, less pronounced than H4Ac, showing, however, the enhanced effect of MeJA over the *axe1-*5 mutation on H3K27me3. Transcription factor families were not significantly overrepresented for *axe1-*5 alone (Fig. 7B). Taken together, MeJA treatment and/or loss of HDA6 were shown to regulate 10.13% of unique TFs by H4ac enrichment and 5.47% of TFs by enrichment of H3K27me3. Interestingly, the comparison of our results with published studies [35, 62] demonstrated that the role of HDA6 extends beyond its directly bound targets at the genome-wide level.

**Table 3:** Transcription factor families significantly represented in H4ac- and H3K27me3-associated AGIs. Statistically significantly under-represented families are shown in italics

Histone modification	TF Family	No. TFs in *A. thaliana*	Condition								
			WT +MeJA			*axe1-*5			*axe1-*5 +MeJA		
			No. of TFs in condition	Percentage representation (%)	*p*-value (Hypergeometric test)	No. of TFs in condition	Percentage representation (%)	*p*-value (Hypergeometric test)	No. of TFs in condition	Percentage representation (%)	*p*-value (Hypergeometric test)
H4ac	AP2	18	4	22.2	2.28E-02	2	11.1	NSS	5	27.8	NSS
	ARR-B	14	0	0.0	NSS	0	0.0	NSS	1	7.1	NSS
	B3	66	3	4.5	NSS	9	13.6	1.67E−02	17	25.8	7.58E−03
	Dof	36	1	2.8	NSS	5	13.9	NSS	11	30.6	8.03E−03
	ERF	122	14	11.5	1.75E−02	9	7.4	NSS	26	21.3	1.57E−02
	HD-ZIP	48	4	8.3	NSS	6	12.5	NSS	11	22.9	NSS
	M-type MADS	66	3	4.5	NSS	11	16.7	1.65E−03	21	31.8	1.37E−04
	MYB	144	10	6.9	NSS	4	2.8	*4.91E*−*02*	14	9.7	NSS
	SRS	11	2	18.2	NSS	0	0.0	NSS	4	36.4	NSS
	ZF-HD	17	2	11.8	NSS	4	23.5	1.69E−02	4	23.5	NSS
	Total	542	43			50			114		
H3K27me3	AP2	18	0	0.0	NSS	0	0.0	NSS	0	0.0	NSS
	ARR-B	14	0	0.0	NSS	1	7.1	NSS	2	14.3	3.85E−02
	B3	66	3	4.5	NSS	0	0.0	NSS	2	3.0	NSS
	Dof	36	2	5.6	NSS	0	0.0	NSS	0	0.0	NSS
	ERF	122	3	2.5	NSS	2	1.6	NSS	4	3.3	NSS
	HD-ZIP	48	2	4.2	NSS	1	2.1	NSS	4	8.3	2.16E−02
	M-type MADS	66	0	0.0	NSS	1	1.5	NSS	3	4.5	NSS
	MYB	144	10	6.9	9.20E−03	1	0.7	NSS	7	4.9	3.96E−02
	SRS	11	2	18.2	4.16E−02	0	0.0	NSS	0	0.0	NSS
	ZF-HD	17	0	0.0	NSS	1	5.9	NSS	2	11.8	NSS
	Total	542	22			7			24		

**Fig. 8:**
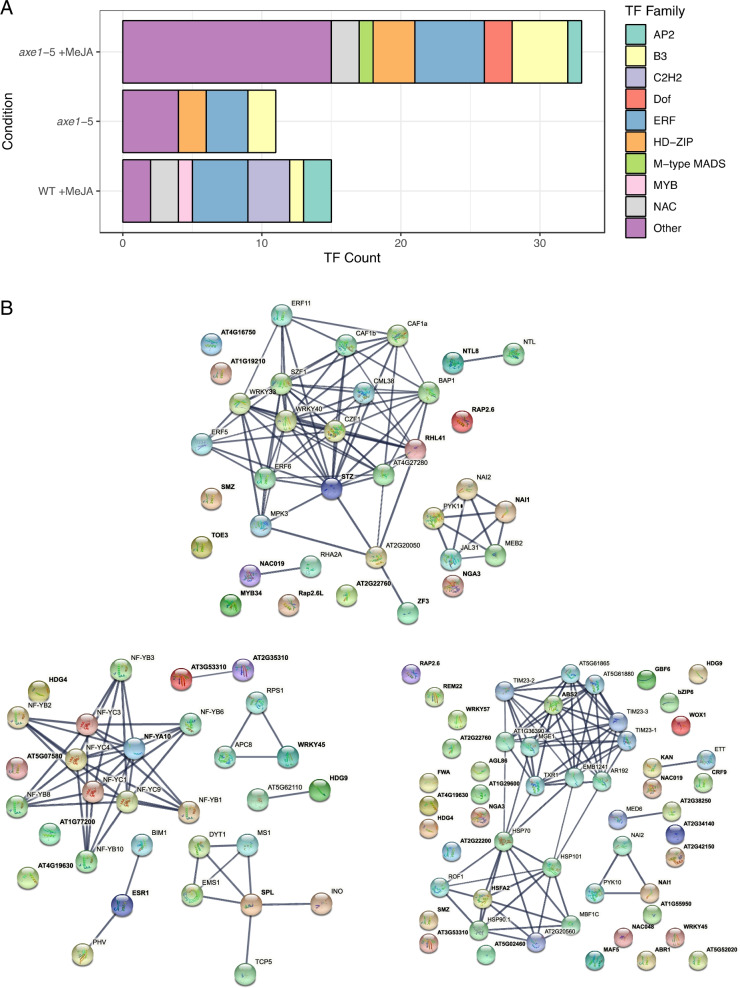
Interaction network of transcription factors enriched in H4ac and transcriptionally upregulated. **A** Counts of transcription factors enriched in H4ac and shown to be transcriptionally induced following microarray analysis. **B** Predictive interaction network inferred by STRING [106] using high-confidence interaction scores (≥ 0.7) with up to 20 interactions shown. The RESPONSIVE TO HIGH LIGHT 41 (RHL41) and SALT TOLERANCE ZINC FINGER (STZ/ZAT10) TFs are central to the WT +MeJA regulatory network, whereas in *axe1-*5 the NUCLEAR FACTOR Y, SUBUNIT A10 (NF-YA10) is central to the largest cluster. The TFs RELATED TO AP2 6 (RAP2.6) and SCHLAFMUTZE (SMZ) were present in both *axe1-*5 +MeJA and WT +MeJA, whilst RAP2.6L was only present in the latter condition. Transcription factors identified as enriched in H4ac and shown to additionally be transcriptionally active following microarray analysis are shown in bold. Statistically significant protein-protein interaction enrichment *p*-values reported (*α* = 0.05). Line thickness is indicative of the strength of data support

A comparison between transcription factors enriched in H4ac and those transcriptionally upregulated in our microarrays was carried out: 15, 11 and 33 TFs were found to be both enriched in H4ac and transcriptionally induced in WT +MeJA, *axe1-*5 and *axe1-*5 +MeJA, respectively [76] (Fig. 8A). To predict downstream interactions and assess the biological relevance of transcriptional networks, the STRING database (v.11.0) [106] was used to investigate putative and experimentally validated interactions of these TFs. The analysis showed a significant enrichment of interactions in the WT +MeJA (*p* < 1.0e−16), *axe1-*5 (*p* < 7.1e−08) and *axe1-*5 +MeJA (*p* < 1.0e−16). At the centre of the WT +MeJA regulatory network (Fig. 8B) are the negative JA biosynthesis regulator STZ/ZAT10, and the stress-responsive RESPONSIVE TO HIGH LIGHT 41 (RHL41/ZAT12) [68, 107]. Conversely, the auxin responder NUCLEAR FACTOR Y, SUBUNIT A10 NF-YA10 TF [108], is central to the network associated with H4ac and transcriptionally induced in *axe1-*5, whilst the ABNORMAL SHOOT 2 (ABS2/NGAL1) and HEAT SHOCK TRANSCRIPTON FACTOR A2 (HSFA2) transcription factors, involved in shoot development and thermotolerance, respectively [109, 110], were central to the *axe1-*5 +MeJA network. Notably, all networks regulated by these TFs are distinct (Fig. 8B).

## Discussion

### A concerted action by methyljasmonate and histone deacetylase 6 on the genome-wide modification of transcriptionally active genes

**Fig. 9:**
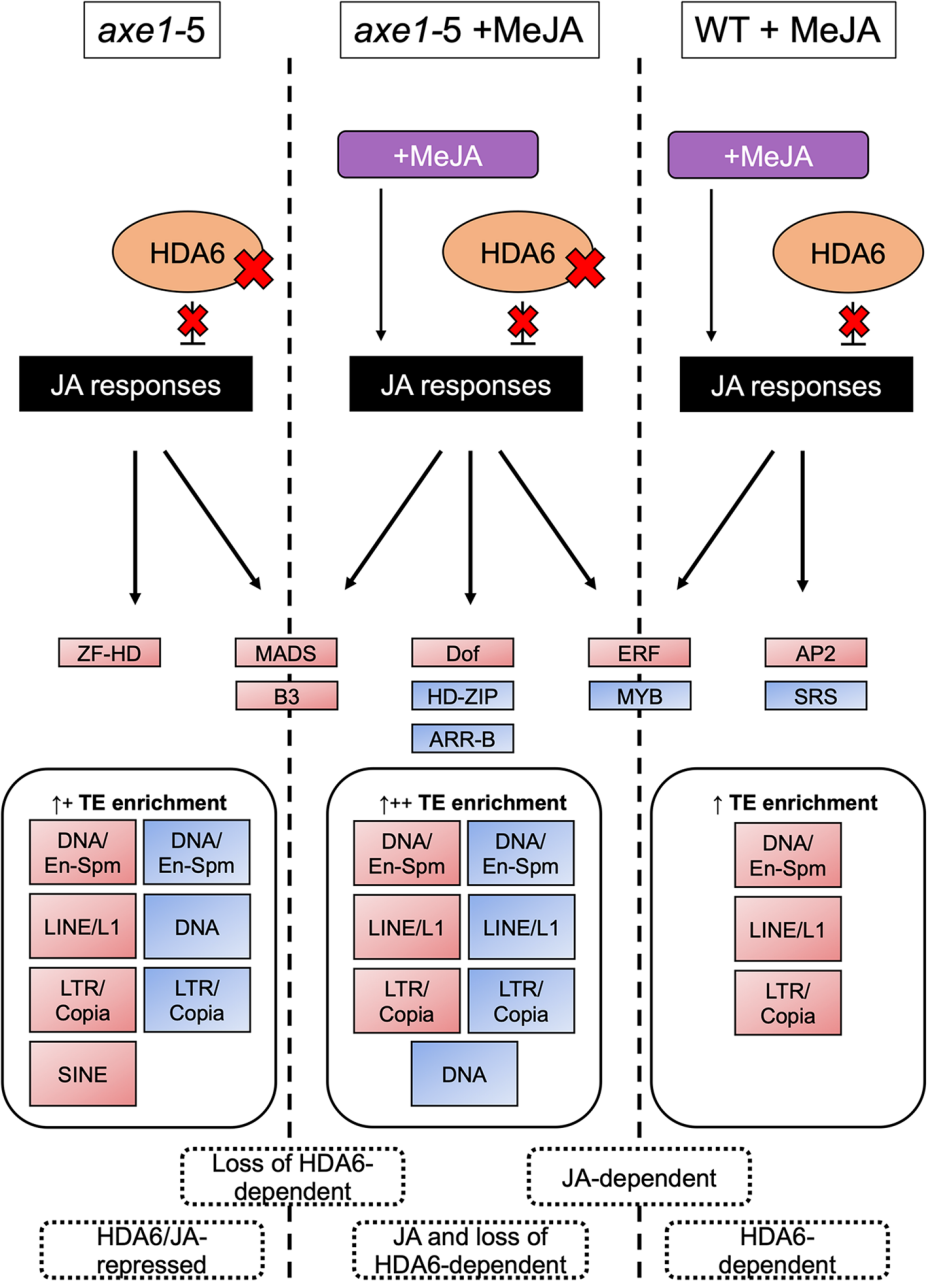
Schematic representation of the responses regulated by histone modifications in WT +MeJA, *axe1-*5 and *axe1-*5 +MeJA conditions. Overrepresented transcription factor families and transposable element (TE) superfamilies are shown in red and blue for enrichment in H4ac and H3K27me3, respectively. Plus (+) symbols indicate relative overrepresentation of TE superfamilies for each condition

Here, we analyse in detail the genome-wide effect of MeJA and HDA6 on histone modifications and highlight novel insights into their differential roles in planta (Fig. 9).

The enrichment of H4ac around the TSS, its lower levels at intergenic regions, and the reduction of H3K27me3 signal around the TSS, and its increase across gene bodies (Fig. 1C) are consistent with previous research [111]. Such observation strengthens the value of our data and the value of its analysis. Collectively, the histone modification distribution observed in *Arabidopsis* faithfully reproduce trends common to other eucaryotes, demonstrating the existence of universally conserved modes of action regulated at the genome level.

The proportion of transcriptionally active genes associated with H4ac was higher in WT +MeJA than in the treated and untreated *axe1-*5 (Fig. 3A), implying that the H4ac enrichment is associated with the JA response and is mediated by HDA6. The absence of HDA6 is known to increase siRNA production, which may explain reduced transcriptional activity in *axe1-*5 [19, 103]. HDA6 has preferential target specificity [31], which may explain the limited unique loci associated with H4ac and H3K27me3. The loss of HDA6 has not been found so far to increase total histone modification, including H4ac [31, 112]. Nevertheless, the reduced association of *axe1-*5 with H4ac and H3K27me3, with respect to WT +MeJA and *axe1-*5 +MeJA (Fig. 3A), provides genome-wide evidence for the specific role of HDA6 on transcriptionally active genes.

Clustering analysis (Fig. 3C) identified groups of genes acetylated either following MeJA treatment or through the absence of HDA6, mimicking the effects of the hormone on the COI1/JAs receptor complex (Clusters IV–VI). This study also uncovers genes enriched in H4ac or H3K27me3 in WT following MeJA treatment (Clusters I and III). In these cases, the enrichment is lost in the absence of HDA6, expanding the HDA6-dependent targets of the JA signalling pathway. Genes uniquely enriched in H4ac in the *axe1-*5 +MeJA (Cluster III) may be deacetylated, hence, repressed, via HDA6 in the presence of MeJA. The clusters identified visually provide novel candidate genes for in planta validation studies examining target binding and nucleosome occupancy, also assigning a fundamental role to JAs on histone modifications and possibly more widely on chromatin remodelling.

### JAs- and HDA6-dependent histone modifications specifically target genes associated with specialized metabolism, as well as abiotic and biotic stress responses

The analysis has identified MeJA-mediated H4Ac enrichment on gene subsets that is either dependent or independent of HDA6 (Fig. 4).

A release of the repressive action of HDA6 is linked to the enrichment of H3K27me3 for specific GO categories associated with defence and immune responses and to the H4ac-associated ‘Plant-pathogen interaction’ KEGG pathway, though these two associations were observed to involve distinct genes. This suggests that histone modifications may regulate gene priming through HDA6 for a more efficient activation of defence responses upon subsequent encounter with pathogens [113].

The JA-inducible JAZ6 and 8 are part of the recently discovered EAR motif-Containing Adaptor Protein (ECAP) adaptor protein [114], recruiting the transcriptional co-repressor, TOPLESS-RELATED 2 (TPR2), into a wider complex that represses the WD-repeat/bHLH/MYB, important activator in the JA-dependent anthocyanin biosynthesis pathway. As TPR2 was not hyperacetylated in our study, it is possible that JA and HDA6 regulate chromatin remodelling through a different mechanism. Remarkably, JAZ4, enriched for H3K27me3 in *axe1-*5 +MeJA, is subjected to an opposite type of regulation. The role of JAZ4 was only recently described, being the only COI1/MYC-independent family member with functions in plant growth and development, supporting the notion that JAZ4-mediated signalling may have distinct branches [115]. Overall, the differential distribution of the two markers on specific targets demonstrates a tight histone modification mechanism employed in the orchestration of JAs signalling, a mechanism that is applied with precision.

The combined effect of HDA6 and MeJA on the H4ac enrichment in ‘Metabolic pathways’ and ‘Biosynthesis of secondary metabolites’ (Fig. 5) highlights the existence of a control mechanism acting on plant metabolism at the genome-wide level. Such control would be fundamental to engage a response to the environment during stress responses.

The regulatory complexity within the JA signalling pathway includes the activation of multiple feedforward and feedback loops [4–6]. The impact of interactions and their kinetics and potency in the wider network are not yet fully understood, and evidence is sparse. Among the seven different branches known for the lipoxygenase (LOX) pathway involved in JA synthesis [116], the allene oxide synthase (AOS) and hydroperoxide lyase (HPL) reactions are concurrent on the product of a 13-LOX enzyme [4]. The HPL branch leads to volatile and non-volatile oxylipin JAs [117]. MYC2 and MYC3 also regulate JA biosynthesis either directly by targeting the JA biosynthesis genes LOX2, 3, 4 and 6 and AOS or indirectly through binding to the AP2-ERF, ORA47 [53, 69]. Significantly, our study shows that HDA6 and MeJA regulate changes that could contribute to the establishment of priming. A change in chromatin structure, due to histone modifications, could facilitate a quicker activation of the JA biosynthesis for a prompt response to stress. This is particularly relevant for a fast activation of the JA pathway during acute response.

MeJA-mediated regulated gene-to-metabolite networks leading to the identification of regulators of nicotine and phenylpropanoid conjugate biosynthesis were identified in *Nicotiana tabacum* cells [118]. Pauwels and Goossens [68] described transcriptional reprogramming simultaneously repressing cell cycle and inducing phenylpropanoid metabolism and production of monolignols and oligolignols, the building blocks of lignin [119] in Arabidopsis cell cultures. The effect of *axe1-*5 on the enrichment for H4ac on ‘Phenylpropanoid biosynthesis’ indicates HDA6 dependency for a module of the pathway for monolignols production (Fig. 6B) ascribing a specific role to HDA6 in dampening activation of this pathway and JA signalling in normal conditions. Above all, MeJA-induced enrichment in H4ac for these enzymes suggests that the oxidative burst after JA perception [120] is regulated at the chromatin level through histone modifications contributing new modes to regulate the plasticity required to trigger a quick response to environmental changes. Importantly, our study also uncovers new regulatory components to the network necessary to store information upon stress exposure.

We previously showed that the translational capacity of cells was affected by MeJA [15] and was associated with leaf size reduction. The MeJA-dependent H4ac enrichment in the GO category ‘ribosome biogenesis’ affects ~10% of the genes transcriptionally upregulated in Noir et al. [15], showing that a subset of these genes involved in acute MeJA responses is targeted specifically by the activating histone marker. The specific association of H4ac with cell cycle regulators, including the ChIP-qPCR validated mitosis-endocycle transition regulators CDKA;1 and E2FB [121], provides evidence for differential histone modification and dependency on MeJA and or HDA6 of these cell cycle regulators that are part of numerous gene families. Whether this is part of a mechanism underlying functional diversification, ultimately contributing to fine-tuning plant developmental plasticity remains to be demonstrated. Our observations highlight specific correlation between MeJA-dependent transcriptional upregulation and H4ac enrichment, providing future opportunities to test whether the genes modified or their targets are directly involved.

### H4ac and H3K27me3 enrichment differentially affects jasmonates and histone deacetylase 6 mediated genome integrity and gene regulatory networks

TEs, affecting genetic variation in plants, are known targets of histone modifying enzymes, including HDA6 targets [31, 98, 100]. The H4ac enrichment observed in *axe1-*5 (Fig. 7A; Table 2), extends the role of HDA6 on TEs [100–102]. LINE/L1 and LTR/COPIA TEs regulate retrogene emergence as a mechanism of phenotypic evolution in Arabidopsis and other dicotyledons [122]. The relationships between TE regulation, MeJA treatment and histone modifications are so far unknown.

The overrepresentation of DNA/En-Spm, LINE/L1 and LTR/COPIA superfamilies followed the loss of HDA6 and MeJA treatment, suggesting that the loss of HDA6, also as part of the JA response, modulates the phenotypic plasticity observed in planta*.* The identification of the expression status of TE in the different conditions will be key for future studies to determine their role. Notably, MeJA treatment alone was also associated with overrepresentation of TEs, demonstrating an unprecedented role for JAs on histone modification.

The loss of HDA6 resulted in the overrepresentation of the non-autonomous SINE transposable elements (also known as CACTA-like transposases) [123] in *axe1-*5 is in line with previous research [32]. This work reinforces the specific role of HDA6 in regulating specific members of the Sadhu TEs. In agreement with To et al. [31], our study identified TE fragments in the SADHU family associated with H4ac in *axe1-*5 irrespective of MeJA, substantiating the role of HDA6 interacting with transposable elements in planta and highlighting further specificity of action as well as novel targets of HDA6 in the context of JA signalling [3]. The *axe1*-5 mediated H3K27me3 and H4ac enrichment of LTR/COPIA would support the interplay between different covalent modifications of the core histone tails [124, 125]. With the ATREP18, DNA superfamily, known to function in DNA repair, replication and recombination [126], being enriched in H3K27me3 in both *axe1-*5 and *axe1-*5 +MeJA, we demonstrate a novel level of regulation operated by JAs that contributes, in synergy with the absence of HDA6 activity, to mobilization of TEs, generating genetic variability to allow the plant to adapt to environmental and developmental challenges.

H4ac and H3K27me3 have distinct roles in governing the MeJA and *axe1-*5-dependent gene regulatory networks (Figs. 7B and 8), acting cumulatively on 24.46% of Arabidopsis transcription factors. JA signalling regulates the trade-off between plant growth and defence mode, with transcription factors being master organizers [6, 53, 69]. Strikingly, MeJA treatment triggered enrichment in H4ac of the AP2 and ERF families, regulating JAs-dependent abiotic stress responses and development [127, 128]. The enrichment in H4ac AP2s like SMZ in all conditions and differential transcriptional MeJA inducibility (Fig. 8B) demonstrates that this type of histone modification depends on either the loss of HDA6 or the presence of JAs, with transcriptional activation being MeJA-dependent. The findings also suggest direct association with JA-mediated H4ac and subsequent transcriptional activation of JA-responsive ERFs [53, 129, 130]. Yu et al. [131] demonstrated that HDA6 interacts with MADS box TFs of the FLOWERING LOCUS C (FLC) clade, including AGAMOUS-LIKE 68, to regulate flowering time in Arabidopsis. We show here that the H4Ac enrichment of members of the JA-inducible M-type MADS, B3 and Dof transcription factor families [132] is mediated not only by HDA6 but also by MeJA (Fig. 7B). This enrichment suggests a role, previously unknown, for JAs on histone modification of specific TFs regulating flowering time. The lack of significant interactions observed through STRING for the 17 TF enriched in H4Ac in *axe1*-5 +MeJA supports the validity of our study in providing a powerful tool to identify novel targets.

MYB21 and 24 associate with JAZs [133, 134] that were enriched in H3K27me3 in the MeJA-treated WT. The repressive H3K27me3 marker was also enriched for the HD-ZIP and the phytohormone crosstalk-associated ARR-B [135] families, whereby MeJA inducibility tracked HDA6 loss. *ARR11* was enriched in H3K27me3 in both treated and untreated *axe1-*5. The *arr11* mutant showed enhanced disease resistance gene induction following MeJA treatment [136]. Therefore, the repressive H3K27me3 marker association with TFs and the variability in MeJA or HDA6 dependency have important downstream implications for transcriptional reprogramming and provide further insight into the role of HDA6 in regulating the JA signalling pathway.

This study advances the understanding of the mechanisms of action through which MeJA or HDA6 contribute to the induction of phenotypic changes in planta. Our network analysis (Figs. 8 and 9) shows that the role of HDA6 in phytohormone crosstalk extends to regulating the H4Ac of specific TFs. The separate networks identified for either WT +MeJA or *axe1-*5 also show the distinct actions of MeJA and HDA6 on TFs. The data available so far in the literature suggest a broader impact of HDA6 on a multitude of environmentally responsive pathways, of which JAs is but one. The overlap between the *axe1*-5 mutant and the *axe1*-5 +MeJA extends beyond transcriptional upregulation as shown by the identification of common TFs between the two interaction networks. It is notable that the predicted interactions of induced TFs were not previously described in the *axe1-*5 +MeJA condition (Fig. 8B).

Taken together, these observations also demonstrate thus-far undescribed functions for the combination of MeJA and HDA6 (Fig. 9). In conclusion, the loss of HDA6 activity and MeJA signalling share functional overlap, albeit maintaining distinct activities on histone modification and, consequently, on chromatin remodelling.

## Conclusions

This high-throughput study shows that the chromatin landscape is modified in Arabidopsis by MeJA and HDA6, alone or in combination, affecting both H4Ac and H3K27me3. The findings suggest the possibility that HDA6-dependent JA regulation can be uncoupled from the wider JA regulatory network, providing novel genome-wide downstream targets for future testing and highlighting the roles of JAs or HDA6 in regulating signalling via histone modifications and possibly more widely on chromatin remodelling (Fig. 9).

A relationship between gene-associated H4ac and MeJA transcriptional upregulation was observed at a genome-wide level. Lack of HDA6 and MeJA treatment showed widespread association with both histone markers. H4ac dynamics were more greatly affected by MeJA treatment and by HDA6 absence. Notably, histone modification has been associated specifically with JAs regulating the activating histone markers on α-linolenic acid metabolism and phenylpropanoid biosynthesis. The effects of HDA6 and MeJA are also associated with transcriptional regulatory hubs for abiotic and biotic stress responses as well as with TE regulation. This study sets the scene for further high-throughput analysis to untangle to what extent specific genes are directly or indirectly affected. Above all, our studies will be invaluable in uncovering targets of MeJA and HDA6 that are regulated at the chromatin level either cooperatively or independently. Thus, regulation could be part of a priming mechanism, also involving metabolic reprogramming, to jump-start the activation of stress responses. In a wider context, the study shows the underlying existence of chromatin remodelling of pathways and regulatory mechanisms controlling enzymes for important plant natural bioproducts that have antioxidant or anti-inflammatory effects for animal health.

## Methods

### Plant materials and growth conditions

Whole *A. thaliana* seedlings of the Histone deacetylase 6 (HDA6) /Reduced potassium dependency 3 (RPD3), *axe1-*5 mutant and its corresponding wild-type DR5 in Col-0 background (namely wild type, WT) lines [29, 31, 39] were grown in vitro on 1% Murashige and Skoog (MS) medium (Duchefa) supplemented with 1% sucrose and 0.8% phytoagar (Duchefa) and buffered to pH 5.8. Stratification of seeds was performed for 3 days at 4 °C in the dark. Seeds were then grown with plates approximately 20° to vertical under 16 h light/8 h dark cycles for 12 days at 22 °C and a light intensity of 100–120 μmol m^−2^s^−1^. Twelve-day-old in vitro grown whole seedlings were transferred to 50 μM MeJA or in parallel to ‘mock’ plates without MeJA and sampled after 3 h for chromatin immunoprecipitation (ChIP) or microarray analysis [76].

### Chromatin immunoprecipitation, library construction, next-generation sequencing and qPCR

ChIP was performed according to Kim et al. [137, 138] on two independent biological replicates. Fresh plants were used without freeze-thawing to prevent the disruption of the protein interactions. Crosslinking of DNA protein was performed prior to sonication. Fragment size range (approx. 200 bp) of the sheared DNA was checked using the Agilent 2100 Bioanalyzer (Agilent Technologies). Antibodies anti-H4 tetra-acetylation (Sigma Aldrich 06-866) and H3K27me3 (Sigma Aldrich 07-449) were used in the immunoprecipitation phase. qPCR analysis of ChIP DNA was performed according to Oszi et al. [139] for three independent biological replicates.

Illumina next-generation sequencing and library construction was delivered via the BBSRC National Capability in Genomics (BB/J010375/1) at Earlham Institute (formerly The Genome Analysis Centre) by Leah Catchpole and Victoria Marshall (Genomics Pipelines Group).

ChIP DNA libraries were prepared according to Illumina TruSeq® ChIP Sample Preparation Kit (Part No: IP-202-1012). The protocol was optimized for 5–10 ng input ChIP DNA with an average final library size of 350–500 bp. DNA was QC’d using PerkinElmer GX High Sensitivity DNA chip (Part No: 5067-4626) and High Sensitivity Qubit assay (part No: Q32854). The insert size of the libraries was verified on an Agilent Technologies 2100 Bioanalyzer using a High Sensitivity DNA chip (Part No: 5067-4627) and concentration determined by using a High Sensitivity Qubit assay. Post-library construction bead-based size selection was performed utilizing Beckman Coulter XP beads (Part No: A63880). Libraries were pooled (eqi-volume) into 2 × 18-plex pools, quantified by qPCR, and run on Illumina HiSeq2500 with a 50-bp single-end rapid run utilizing v2 chemistry aiming for 13 million reads per sample.

SOLiD library preparation and pair end sequencing were performed by the Genome Network Analysis Support Facility, RIKEN CLST as previously described [138, 140, 141].

Primers used in qPCR experiments (5′–3′):

**Table:** 

AT1G27730	ZAT10-CHIP-F	TCACACGTTTGCACCATCTG
AT1G27730	ZAT10-CHIP-R	CGGAGTTGGACACGCTACTA
AT3G18780	Actin2_qRT_F	CGCTGACCGTATGAGCAAAG
AT3G18780	Actin2_qRT_R	TTCATGCTGCTTGGTGCAA
AT3G48750	CDKA-1-CHIP-F	CGTCGGTGTGCTAGTCTCA
AT3G48750	CDKA-1-CHIP-R	AAACAAGTTCCTCCTCCGGA
AT5G13330	RAP2.6L-CHIP-F	GGTATGGAAAGGGACCGGTT
AT5G13330	RAP2.6L-CHIP-R	CGGGTCTGTCGGATTCTCTA
AT5G22220	E2FB-CHIP-F	AGATCCGAGTTTCCGTGACA
AT5G22220	E2FB-CHIP-R	TGCCGCTTAGAAGATGGGAA
AT5G67300	MYB44-CHIP-F	GAATCACTGGAACTCGACGC
AT5G67300	MYB44-CHIP-R	CCGCACTCACCGATCTCTTA

Primers were designed in correspondence of the genomic regions most highly enriched for histone markers.

### ChIP-Seq data processing

Reads from demultiplexed fastq data files were trimmed using the sliding window operation from Trimmomatic (version 0.36.5) [142] by averaging the quality of reads across four bases, with a quality score threshold of 20. The outputs were checked using the FASTQC and MultiQC tools [143, 144] and were all high quality (Additional file 1: Figure S1). Reads were aligned to the TAIR10 assembly of the *A. thaliana* genome with Bowtie for Illumina [145, 146] using default parameters.

Peak calling of ChIP-enriched fragments was performed using MACS (version 1.4.2) [147] against input reads (Supplemental Figure 2). The choice of MACS version was justified by the findings of Jeon et al. [148]. The effective genome size was set to 1.35e+08 [149] with lower and upper mfold bound limits of 5 and 50, respectively. The genomic features associated with ChIP-Seq peaks were annotated using PAVIS (Additional file 1: Figure S2) [150], with up- and downstream regions designated as falling within a distance of 2000 bp from the TSS or TTS, respectively. ChIP enrichment signal across genes was obtained by calculating the log2 ratio of ChIP:input signal and associated with gene regions using the bamCompare and computeMatrix tools, respectively, from the deepTools2 package [74].

Peak summit loci were cross-referenced with TAIR10, and genes or transposable elements were deemed significant in a pre-determined condition if the peak summit was present in the intragenic region. To obtain a list of genes or transposable elements deemed to have undergone differential H4ac or H3K27me3-associated modifications with respect to the untreated WT control, genes that were only associated with peaks in the conditions of interest, or where the peak enrichment was twofold with respect to the control, were reported. Genes meeting these criteria in both biological replicates were used. Data from Hung et al. [35, 62] (GSE132563, GSE132636 and GSE133005) was analysed using the same methodology. AGI subsets corresponding to transcription factors were identified using the Plant Transcription Factor Database [104].

Analysis of overrepresented GO terms was conducted in the BiNGO plugin for Cytoscape (v.3.6.1) [81, 82] using a hypergeometric test for overrepresentation and a Benjamini-Hochberg false discovery rate (FDR) correction (*α* = 0.05) [151] and redundant terms consolidated using [83]. Overrepresentation of KEGG pathways was carried out using DAVID [86, 87]. Overrepresentation of KEGG pathways was calculated using Fisher’s exact test in DAVID [86, 87]. Hypergeometric overrepresentation testing of transcription factor families and transposable elements superfamilies was performed in R.

### Microarray analysis

cDNA synthesis was carried out using 500 ng of total RNA (RNAeasy, QIAGEN) and colour labelled with Cy3 using the Quick Amp labelling kit from Agilent Technologies. This was followed by fragmentation and hybridisation to the Arabidopsis oligo DNA microarray version 4.0 (Agilent Technologies). Three biological replicates were used for each.

Arrays were scanned with a microarray scanner (G2505B, Agilent Technologies) and analysed using GeneSpring version7 (Agilent Technologies) according to To et al. [31]. Raw signals less than 0.01 were adjusted to 0.01 and a 75 percentile normalization was performed for each chip. Genes with at least a 2-fold difference in their expression levels were evaluated with Student’s *t* test and genes with *p*-values < 0.05 were considered to be differentially expressed in *axe1-*5 and/or with MeJA treatment when compared to the untreated control.

All analyses subsequent to the raw data pre-processing and normalization were performed in MATLAB. Gene expression values flagged as ‘Absent’ or with missing in at least one replicate were removed from the dataset and Agilent probe names were mapped to AGIs and gene annotation retrieved. Differentially expressed genes were identified by using Student’s *t* test between the conditions of interest using the *ttest2* function in MATLAB. The *p*-values obtained from the *t*-test were corrected for multiple testing by calculating the estimated false discovery rates (FDR). The FDR was estimated using the Benjamini-Hochberg method [151] and was implemented using the *mafdr* function in MATLAB. The FDR was set to 0.05 and genes below this threshold were differentially expressed. Genes that were up- or downregulated between the conditions of interest were identified by right- or left-tailed *t*-tests, respectively [76].

### Transcription factor interaction network prediction

In silico prediction of transcription factor interactions was carried out using the STRING database [106]. High-confidence interaction scores (≥ 0.7) were reported for all interaction sources (textmining, experiments, databases, co-expression, neighbourhood, gene fusion and co-occurrence) and statistically significant protein-protein interaction enrichment *p*-values reported (*α* = 0.05).

## Supplementary Information

**Additional file 1: Figure S1.** Quality control of sequencing reads and ChIP-Seq peaks. **Figure S2.** MACS peak models. **Figure S3.** ChIP-Seq data sample similarity. **Figure S4.** Principal component analysis. **Figure S5.** H4ac- and H3K27me3-associated genes identified by SOLiD and Illumina. **Figure S6.** Distribution of enriched ChIP-Seq peaks across genomic features. **Figure S7.** Secondary metabolite biosynthesis pathways mapped to conditions of interest using iPath3.

**Additional file 2: Table S1.** Distribution of histone modification-associated peaks across genomic regions. **Table S2.** Differential enrichment (fold change) of histone modification-associated peak summits in condition of interest compared to untreated WT. **Table S3.** Overlap of AGIs enriched in H4ac and H3K27me3 in *axe1-*5 with H3ac, H3K4me2 and HDA6 ChIP-Seq by Hung et al. (2019; 2020). **Table S4.** List of Gene Ontology terms overrepresented by AGIs associated with H4ac or H3K27me3. **Table S5.** Overrepresentation of transposable element superfamilies in H4ac- and H3K27me3-associated loci. **Table S6.** Overrepresentation of transcription factor families in H4ac- and H3K27me3-associated AGIs.

**Additional file 3: Supplementary Data Table.** List of genes enriched in H4ac or H3K27me3 in all conditions of interest. Annotation from TAIR 10.

## Data Availability

The datasets supporting the conclusions of this article are included within the article and its additional files. The ChIP-Seq dataset is available in the GEO repository (GEO ID: GSE 197733) [70]. The microarray dataset is available in the GEO repository (GEO ID: GSE162891) [76].

## Abbreviations

ABA: Abscisic acid
ABS2: ABNORMAL SHOOT 2
AOS: Allene oxide synthase
AP2: APETALA2
ARR-B: ARABIDOPSIS RESPONSE REGULATOR-B
B3: B3 DOMAIN
bHLH: Basic helix-loop-helix
BP: Biological process
CC: Cellular component
CDKA: CYCLIN-DEPENDENT KINASE A
ChIP: Chromatin immunoprecipitation
COI1: CORONATINE INSENSITIVE1
Dof: DNA-binding with one finger
ECAP: EAR motif-Containing Adaptor Protein
EIL1: ETHYLENE-INSENSITIVE3-like
EIN3: ETHYLENE-INSENSITIVE3
ERF: Ethylene responsive element binding factor
GA: Gibberellic acid
GID1a: GA-INSENSITIVE DWARF1a
GO: Gene ontology
H3ac: Histone 3 acetylation
H3K27me3: Histone 3 lysine 27 trimethylation
H3K4me2: Histone 3 lysine 4 dimethylation
H4ac: Histone 4 acetylation
HAT: Histone acetyltransferase
HD-ZIP: HOMEODOMAIN-LEUCINE ZIPPER
HDA6: Histone deacetylase 6
HDAC: Histone deacetylase
HDM: Histone demethylase
HPL: Hydroperoxide lyase
HSFA2: HEAT SHOCK TRANSCRIPTON FACTOR A2
JA: Jasmonate
JA-Ile: Jasmonoyl-L-isoleucine
JAZ: JASMONATE ZIM-DOMAIN
LINE: Long interspersed elements
LOX: Lipoxygenase
LTR: Long terminal repeat
MeJA: Methyl jasmonate
MF: Molecular Function
NF-YA10: NUCLEAR FACTOR Y, SUBUNIT A10
NINJA: Novel interactor of JAZ
qPCR: Quantitative polymerase chain reaction
RAP2.6: RELATED TO AP2 6
RHL41: RESPONSIVE TO HIGH LIGHT 41
RPD3b: Reduced Potassium Dependency 3-like
SEM: Standard error of the mean
SINE: Short interspersed elements
SOLiD: Sequencing by Oligonucleotide Ligation and Detection
SRS: SERYL-TRNA SYNTHETASE
STZ: SALT TOLERANCE ZINC FINGER
TE: Transposable element
TF: Transcription factor
TPL: TOPLESS
TSS: Transcription start site
TTS: Transcription termination site
WT: Wild type
ZF-HD: ZINC FINGER HOMEODOMAIN
