# Supplementary material for: Jasmonates and Histone deacetylase 6 activate Arabidopsis genome-wide histone acetylation and methylation during the early acute stress response
Source: BMC Biol. 2022 Apr 11;20:83. doi: 10.1186/s12915-022-01273-8 (PMC8996529; doi:10.1186/s12915-022-01273-8)
Supplement: Supplementary file 1 — Additional file 1: Figure S1. Quality control of sequencing reads and ChIP-Seq peaks. Figure S2. MACS peak models. Figure S3. ChIP-Seq data sample similarity. Figure S4. Principal component analysis. Figure S5. H4ac- and H3K27me3-associated genes identified by SOLiD and Illumina. Figure S6. Distribution of enriched ChIP-Seq peaks across genomic features. Figure S7. Secondary metabolite biosynthesis pathways mapped to conditions of interest using iPath3. [file 12915_2022_1273_MOESM1_ESM.pdf]

Manuscript Title:

Jasmonates and Histone deacetylase 6 activate Arabidopsis genome-wide histone acetylation and methylation during the early acute stress response

Stacey Vincent[1], Jong-Myong Kim[2,4], Imma Pérez-Salamó[1], Taiko Kim To[2,5], Chieko Torii[2], Junko Ishida[2], Maho Tanaka[2], Takaho A. Endo[3,6], Prajwal Bhat[1], Paul Devlin[1], Motoaki Seki[2,*] and Alessandra Devoto[1*]

Information on Additional Information

Additional File 1: Figures S1-S6

Figure S1. Quality control of sequencing reads and ChIP-Seq peaks.

Figure S2. MACS peak models.

Figure S3. ChIP-Seq data sample similarity

Figure S4. Principal component analysis

Figure S5. H4ac- and H3K27me3-associated genes identified by SOLiD and Illumina

Figure S6. Distribution of enriched ChIP-Seq peaks across genomic features

Figure S7. Secondary metabolite biosynthesis pathways mapped to conditions of interest using iPath3.

Supplemental Figures

Figure S1

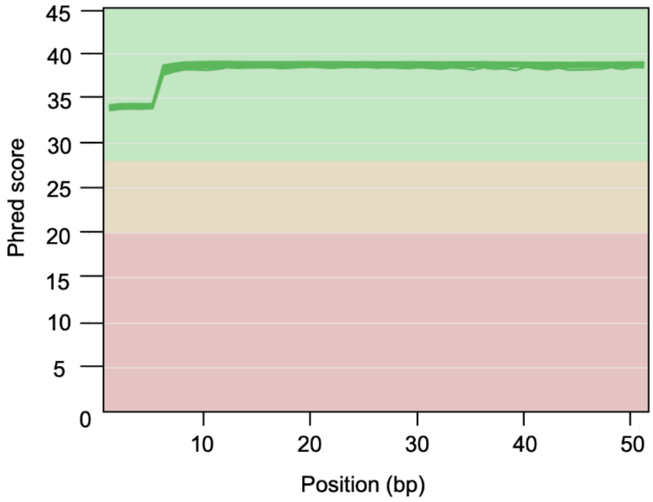

Figure S1. Quality control of sequencing reads and ChIP-Seq peaks. A. Average Phred quality score (FastQC: mean of quality scores) of all samples generated on the Illumina HiSeq platform. Quality scores obtained using FastQC and aggregated using MultiQC after trimming using Trimmomatic (Andrews, 2010; Bolger et al., 2014; Ewels et al., 2016; Afgan et al., 2018).

Figure S2

A

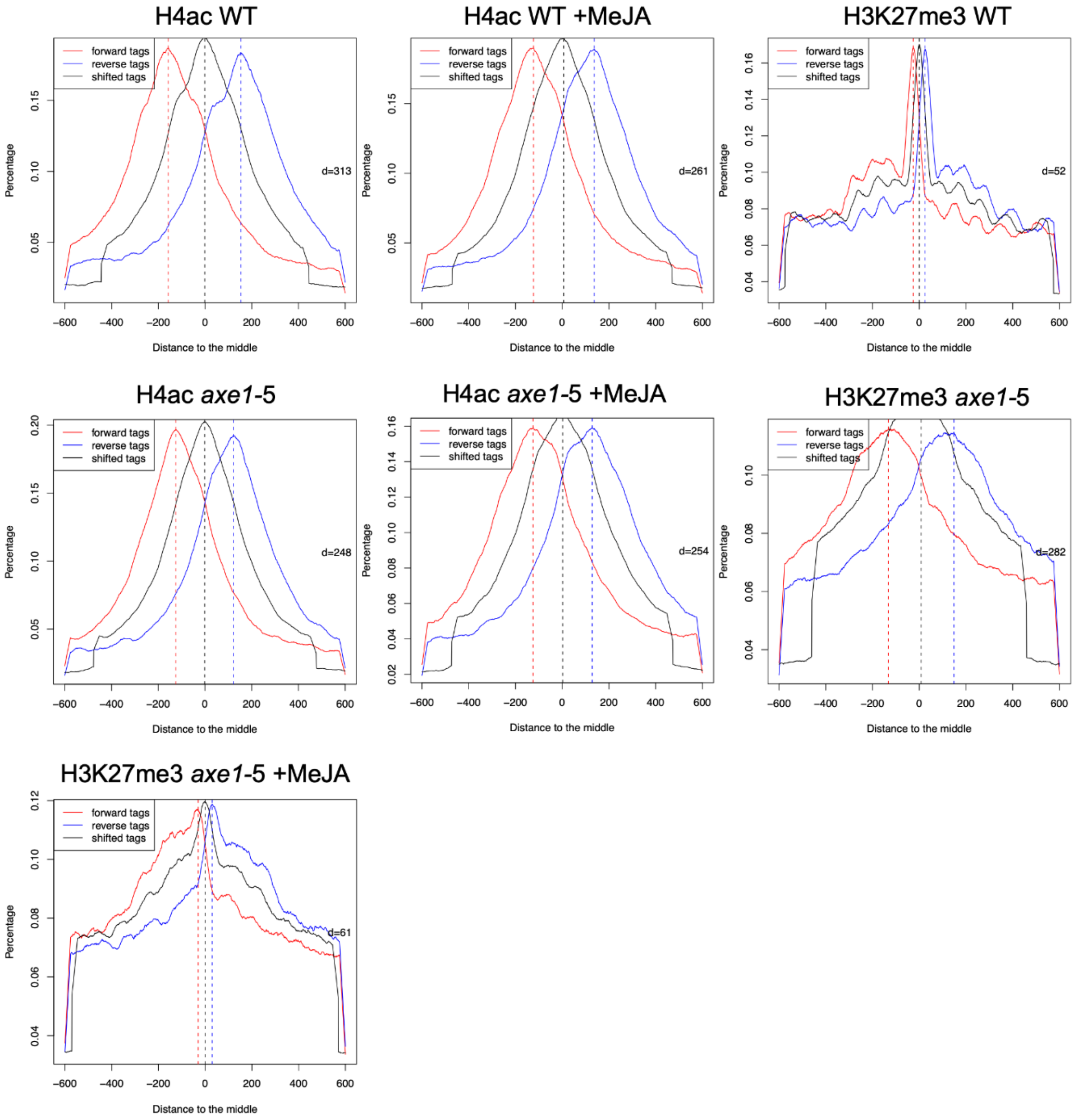

H4ac WT
H4ac WT +MeJA
H3K27me3 WT
H4ac *axe1*-5
H4ac *axe1*-5 +MeJA
H3K27me3 *axe1*-5
H3K27me3 *axe1*-5 +MeJA
forward tags
reverse tags
shifted tags
Percentage
Distance to the middle
d=313
d=261
d=52
d=248
d=254
d=282
d=61

Figure S2

B

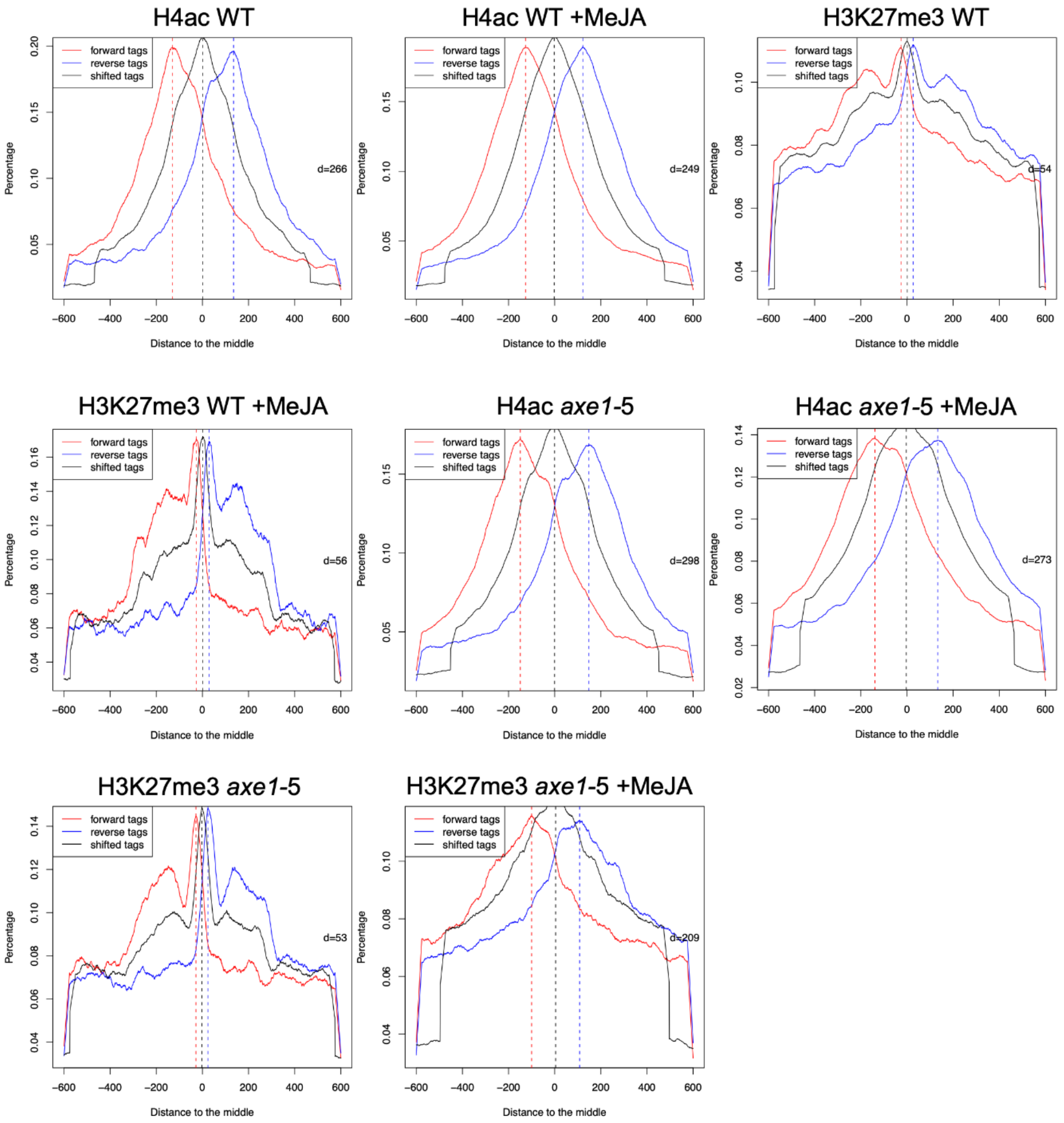

Figure S2. MACS peak models. Peak model generated on H4ac and H3K27me3 ChIP-Seq reads after peak calling using MACS (v. 1.4.2) [4, 19]. Tag shift after peak calling for both strands is shown for Replicate 1 (A) and 2 (B).

Figure S3

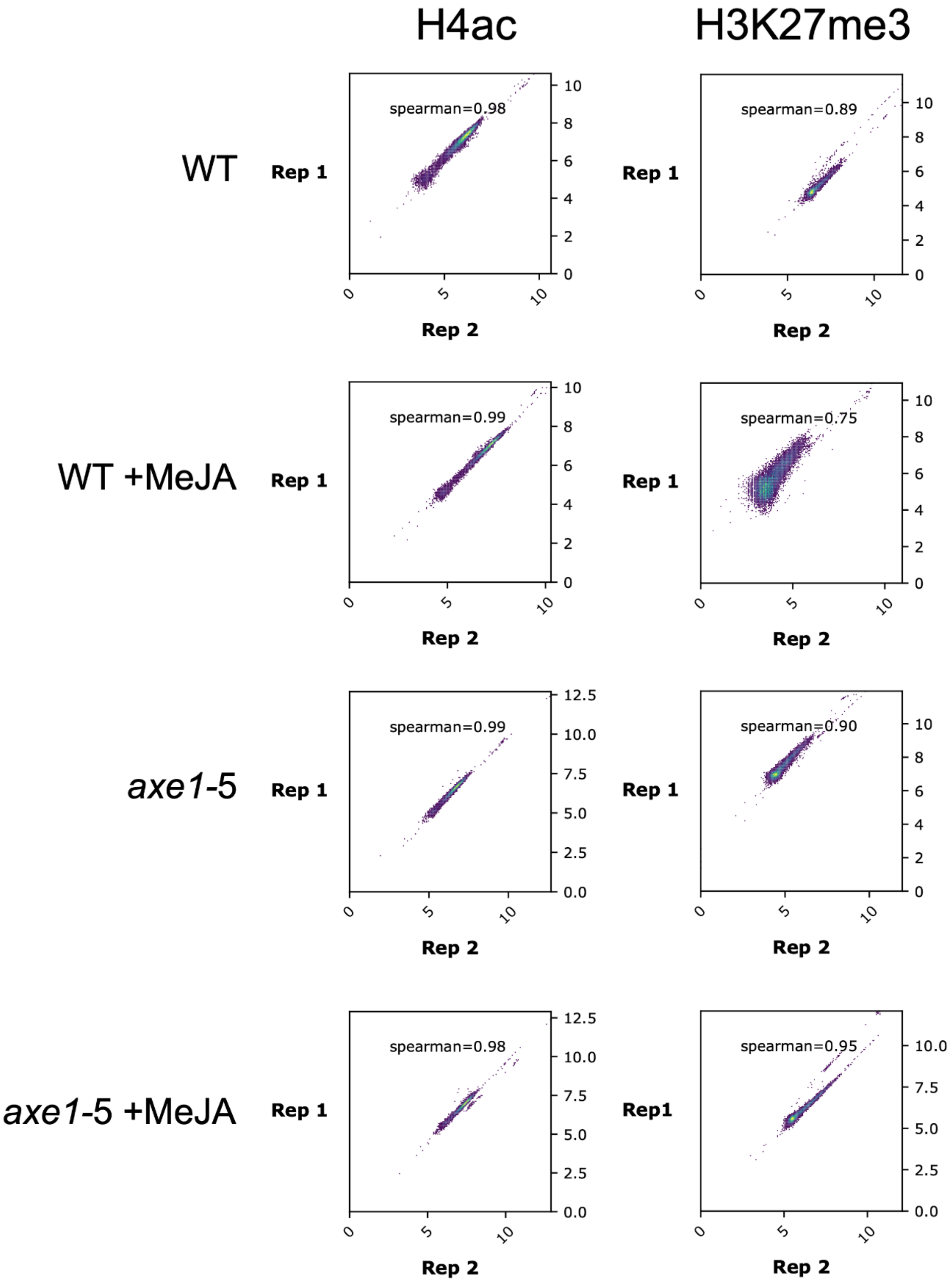

Figure S3. ChIP-Seq data sample similarity. Spearman's rank correlation between all ChIP-Seq replicates for the H4ac and H3K27me3 histone markers or between Illumina and SOLiD samples (WT +MeJA, H3K27me3). All scatter plots of values of Illumina replicates showing high correlation.

Figure S4

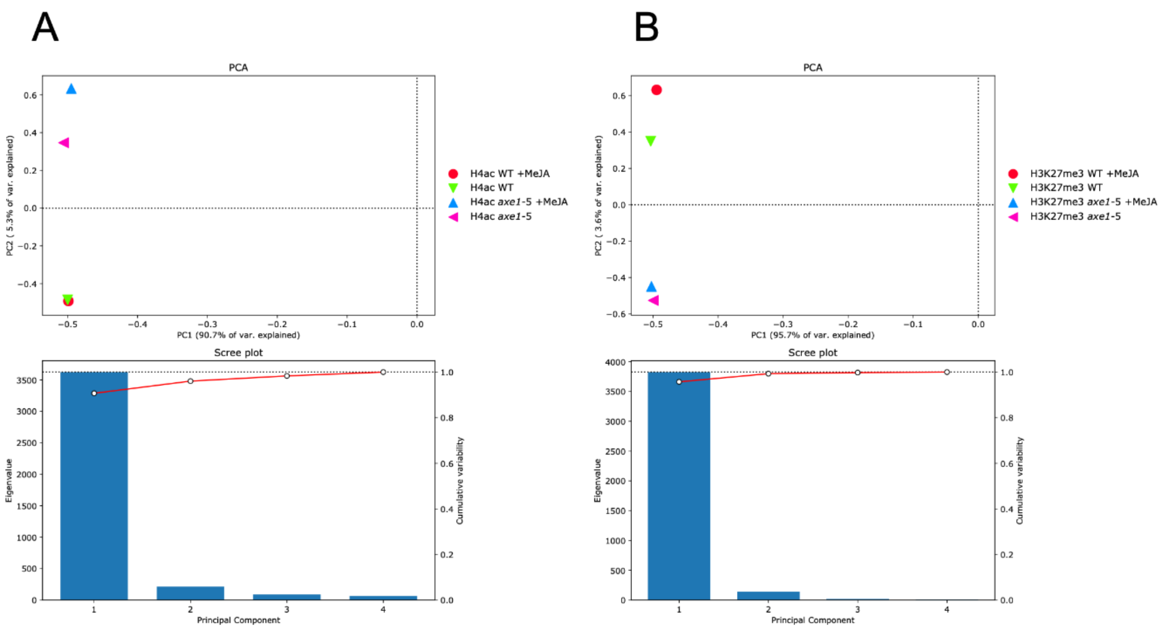

Figure S4. Principal component analysis. PCA and scree plots of ChIP-Seq WT and *axe1-5* samples +/- MeJA relative to input DNA for H4ac (A) and H3K27me3 (B) Data is shown for the first and second principal components for one representative replicate. Ordination analysis was calculated for ChIP tag pileup for aligned sequences.

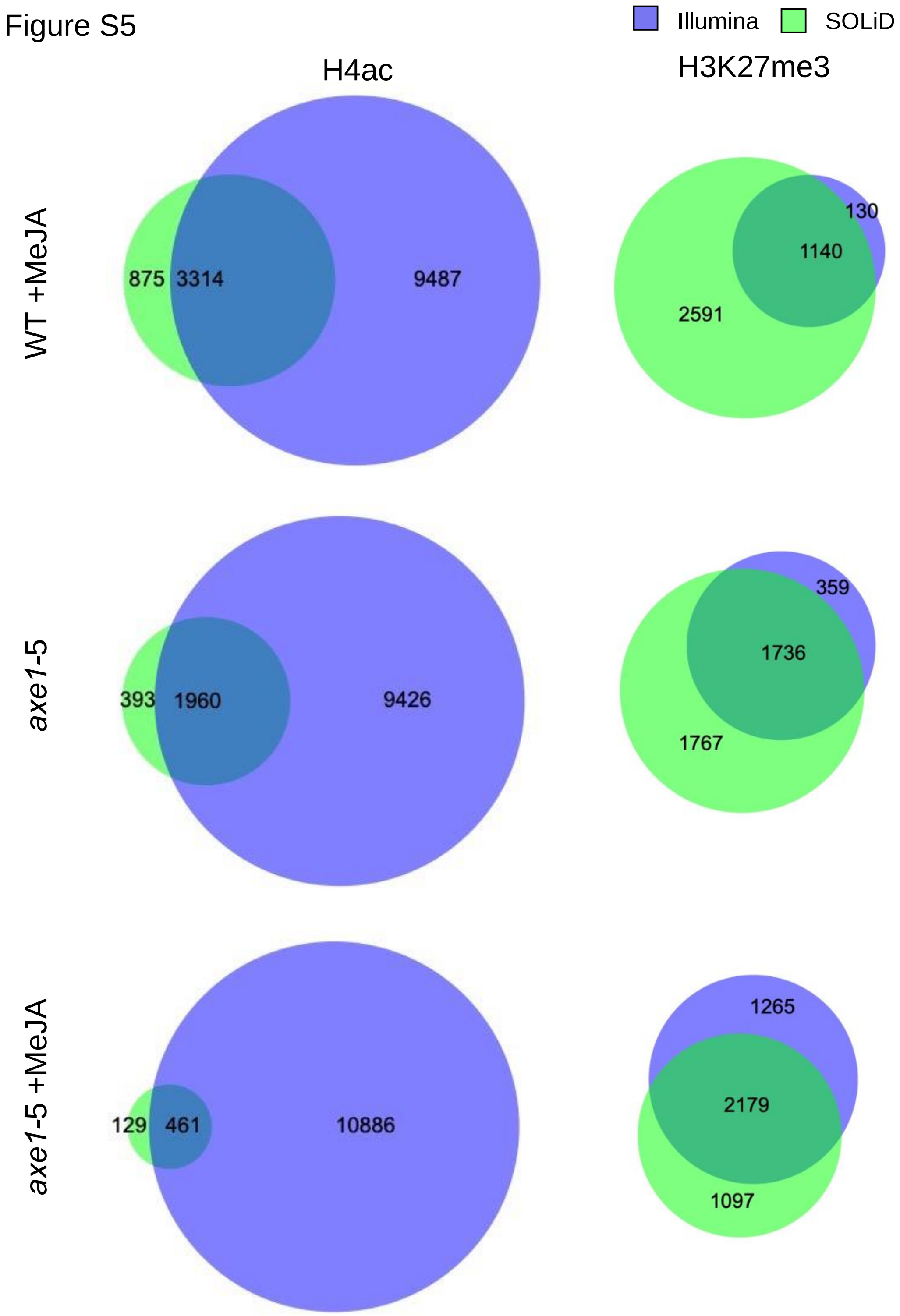

Figure S5. H4ac- and H3K27me3-associated genes identified by SOLiD and Illumina. Comparison of H4ac- and H3K27me3-associated genes identified following ChIP-Seq analysis in three conditions of interest on the Illumina and SOLiD high-throughput sequencing platforms. Genes were considered significant in each condition if the peak summit generated by MACS was situated within an identified gene. For Illumina, N=2 (except in WT +MeJA, N=1) and in SOLiD N=1. Diagrams created in BioVenn [22].

Figure S6

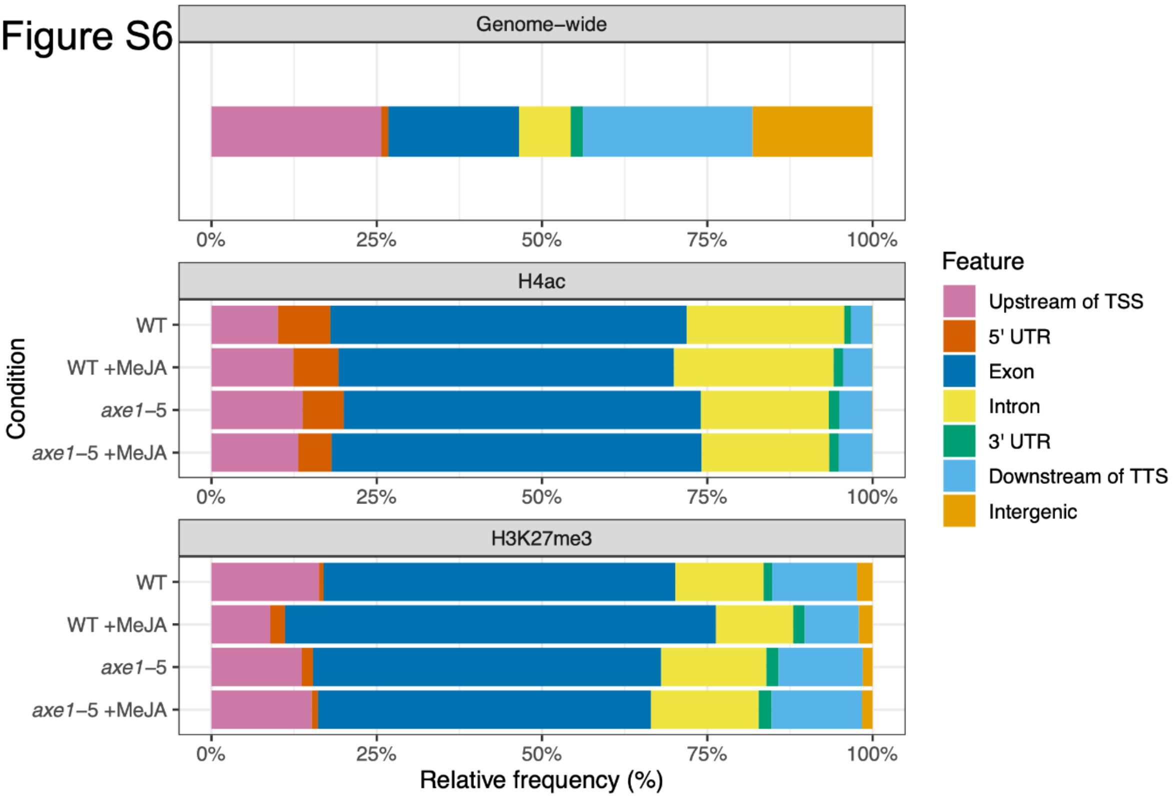
Genome−wide
0%
25%
50%
75%
100%
H4ac
Condition
WT
WT +MeJA
axe1−5
axe1−5 +MeJA
H3K27me3
Relative frequency (%)
Feature
Upstream of TSS
5' UTR
Exon
Intron
3' UTR
Downstream of TTS
Intergenic

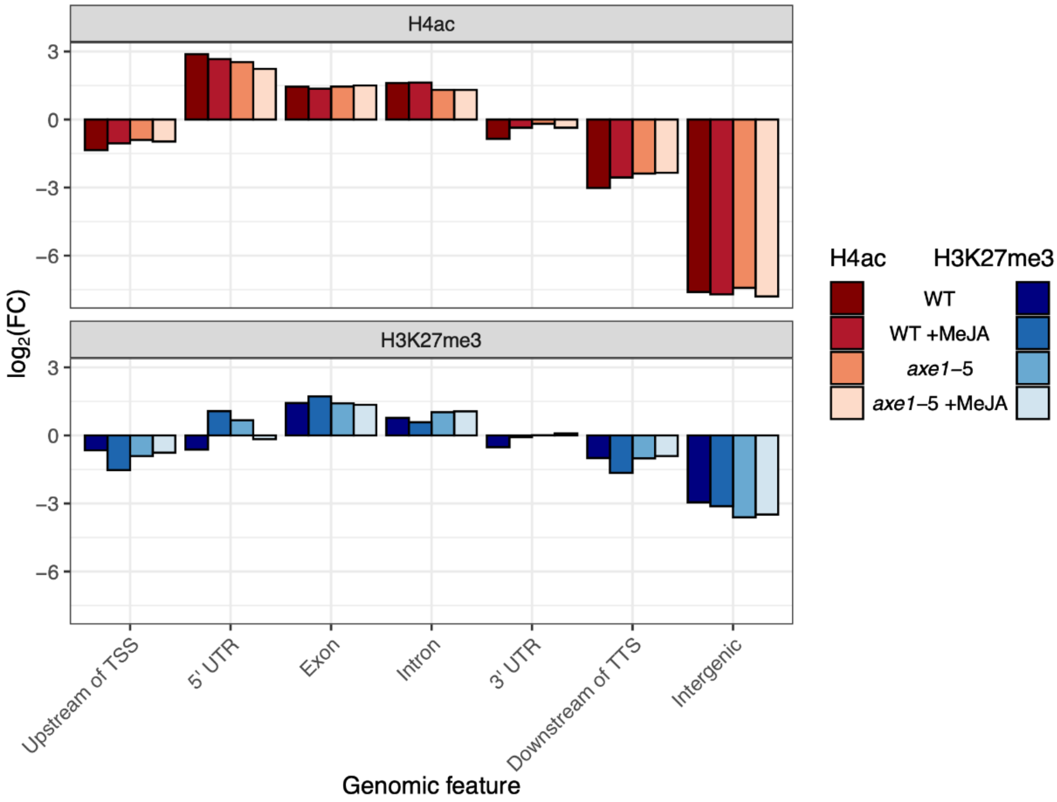
H4ac
H3K27me3
log2(FC)
3
0
−3
−6
Upstream of TSS
5' UTR
Exon
Intron
3' UTR
Downstream of TTS
Intergenic
Genomic feature
WT
WT +MeJA
axe1−5
axe1−5 +MeJA

Supplemental Figure 6. Distribution of enriched ChIP-Seq peaks across genomic features. A. Relative frequency of H4ac or H3K27me3 distribution with respect to % representation in genomic regions (TAIR10), upstream of the TSS; 5' untranslated region (UTR); exons; introns; 3' UTR; downstream of the TTS and intergenic region, in WT and *axe1*-5 +/- MeJA treatment. Peaks generated using Model-based Analysis of ChIP-Seq (MACS) (version 1.4.2) were annotated using PAVIS [4, 7].

B. Fold change (log2) of relative frequency of genomic features in ChIP-Seq samples

Figure S7

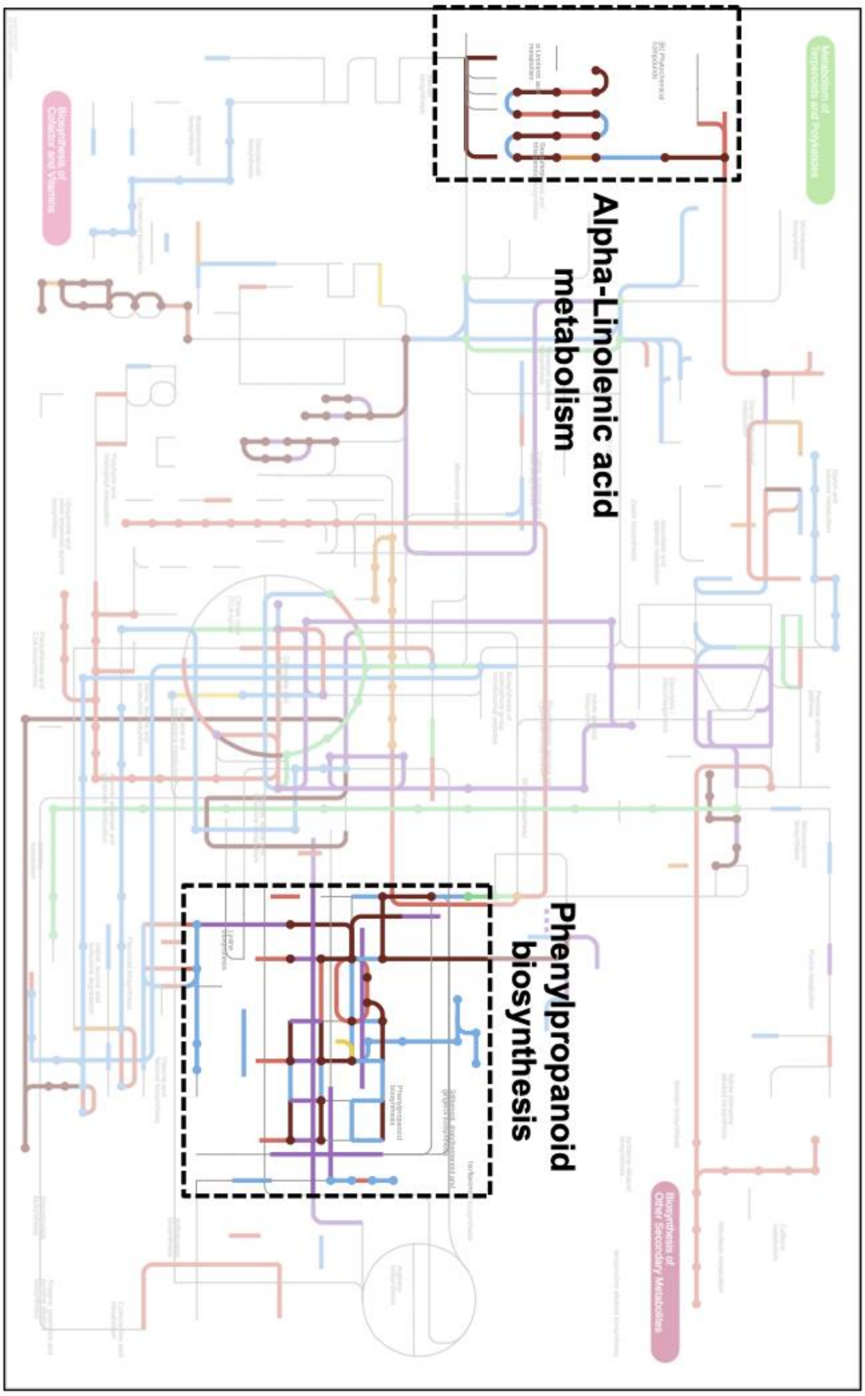

Figure S7. Mapping secondary metabolite biosynthesis pathways onto Arabidopsis metabolism for the conditions of interest using iPath3 (Darzi et al., 2018). Colour set to reflect condition of interest (blue: WT +MeJA; yellow: *axe1*-5; red: *axe1*-5+MeJA for H4ac associated genes. Combination of primary colours indicates overlap between conditions (green: WT +MeJA and *axe1*-5; orange: *axe1*-5 and *axe1*-5 +MeJA; purple:WT +MeJA and *axe1*-5 +MeJA; brown: present in all 3 conditions). Dots represent metabolites and lines

represents enzymes acting on them. Insets: α -linolenic acid metabolism (α-LA) and (B) phenylpropanoid (PP) biosynthesis described in Figure 6.
